# Supplementary material for: Methodological considerations for linking household and healthcare provider data for estimating effective coverage: a systematic review
Source: BMJ Open. 2021 Aug 26;11(8):e045704. doi: 10.1136/bmjopen-2020-045704 (PMC8395298; doi:10.1136/bmjopen-2020-045704)
Supplement: Supplementary data [file bmjopen-2020-045704supp001.pdf]

Supplemental File 1: Medline Search Terms

Term 1: Effective coverage (including related terms e.g. benchmarking, systems dynamics)

Term 2: Linked population-based and health provider analyses

Term 3: Population-based survey validity

Term 4: Health provider quality of care measurement

Term 5: Geospatial measurement of health provider utilization

Term 6: Date and Medline restriction

(Term 1 -OR- Term 2 -OR- Term 3 -OR- Term 4 -OR- Term 5) -AND- Term 6

Term 1:

(((coverage[Title/Abstract]) NOT (insurance[Title/Abstract])) AND (benchmark*[Title/Abstract])) OR (((coverage[Title/Abstract]) NOT (insurance[Title/Abstract])) AND (("systems dynamic framework"[Title/Abstract]) OR ("system dynamics framework"[Title/Abstract]) OR ("systems dynamics framework"[Title/Abstract]))) OR (((“universal health coverage”[Title/Abstract]) OR (UHC[Title/Abstract]) OR (“universal health care”[Title/Abstract])) AND ((coverage[Title/Abstract]) NOT (insurance[Title/Abstract])) AND ((metric*[Title/Abstract]) OR (measur*[Title/Abstract]) OR (score*[Title/Abstract]) OR (indicator*[Title/Abstract]) OR (variable*[Title/Abstract]) OR (index[Title/Abstract]) OR (indices[Title/Abstract]))) OR (("quality-adjusted") AND ((coverage) NOT (insurance[Title/Abstract]))) OR (("effective coverage"[Title/Abstract]) NOT (insurance[Title/Abstract])) OR

Term 2:

((((coverage) NOT (insurance)) AND ((link*[Title/Abstract]) OR (match*[Title/Abstract]) OR (combine*[Title/Abstract]) OR (merge*[Title/Abstract]) OR (attach*[Title/Abstract]) OR (join*[Title/Abstract]) OR (pair*[Title/Abstract]) OR (connect*[Title/Abstract]))) AND (("access to care") OR ("service quality") OR (quality of health care[MeSH Terms]) OR ("service readiness") OR ("service provision") OR ("service delivery") OR ("source of care") OR ("where care was sought") OR ("facility survey") OR ("provider survey") OR ("facility assessment") OR ("provider assessment") OR ("facility data") OR ("provider data") OR ("outlet survey") OR ("outlet assessment") OR ("outlet data")) AND (("service use") OR ("service utilization") OR ("care seeking") OR ("care-seeking") OR ("careseeking") OR ("doctor visit") OR ("clinic visit") OR ("facility visit") OR ("household survey") OR ("household data") OR ("household assessment") OR ("population survey") OR ("demographic survey") OR ("demographic and health survey") OR ("population-based"))) OR

Term 3:

((((("self report"[Title/Abstract]) OR ("self-report"[Title/Abstract]) AND (((maternal[Title/Abstract]) OR (newborn[Title/Abstract]) OR (child[Title/Abstract])) AND (health[Title/Abstract]))) OR ("maternal report"[Title/Abstract]) OR ("maternal recall"[Title/Abstract]) OR ("caregiver recall"[Title/Abstract]) OR ("caregiver report"[Title/Abstract]))) AND ((concordance[Title/Abstract]) OR (accuracy[Title/Abstract]) OR (valid*[Title/Abstract]))) OR

Term 4:

((((“structural quality”[Title/Abstract]) NOT (“structural equation”[Title/Abstract])) OR (infrastructur*[Title/Abstract]) OR (input*[Title/Abstract]) OR (readiness[Title/Abstract]) OR (capacity[Title/Abstract]) OR (“service provision”[Title/Abstract]) OR (“service environment”[Title/Abstract])) AND ((“quality of care”[Title/Abstract]) OR (process[Title/Abstract]) OR (“content of care”[Title/Abstract]) OR (“health outcome”[Title/Abstract]) OR (“health outcomes”[Title/Abstract]) OR ((“population health”[Title/Abstract]) AND (outcome*[Title/Abstract]))) AND ((metric*[Title/Abstract]) OR (measur*[Title/Abstract]) OR (indicator*[Title/Abstract]) OR (score*[Title/Abstract]) OR (variable*[Title/Abstract]) OR (index[Title/Abstract]) OR (indices[Title/Abstract])) AND (survey[Title/Abstract])) OR

Term 5:

((provider[Title/Abstract]) OR (facility[Title/Abstract]) OR (hospital[Title/Abstract]) OR ("source of care"[Title/Abstract])) AND ((geographic[Title/Abstract]) OR (spatial[Title/Abstract]) OR (GIS[Title/Abstract])) AND ((measur*[Title/Abstract]) OR (model*[Title/Abstract])) AND ((utilization[Title/Abstract]) OR ("usage"[Title/Abstract]) OR (distance[Title/Abstract]))) AND

Term 6:

(medline[sb]) AND ("2000/01/01"[PDat] : "2021/03/29"[PDat])
