# Supplementary material for: Methodological considerations for linking household and healthcare provider data for estimating effective coverage: a systematic review
Source: BMJ Open. 2021 Aug 26;11(8):e045704. doi: 10.1136/bmjopen-2020-045704 (PMC8395298; doi:10.1136/bmjopen-2020-045704)
Supplement: Author's manuscript [file bmjopen-2020-045704.draft_revisions.pdf]

BMJ Open is committed to open peer review. As part of this commitment we make the peer review history of every article we publish publicly available.

When an article is published we post the peer reviewers' comments and the authors' responses online. We also post the versions of the paper that were used during peer review. These are the versions that the peer review comments apply to.

The versions of the paper that follow are the versions that were submitted during the peer review process. They are not the versions of record or the final published versions. They should not be cited or distributed as the published version of this manuscript.

BMJ Open is an open access journal and the full, final, typeset and author-corrected version of record of the manuscript is available on our site with no access controls, subscription charges or pay-per-view fees (http://bmjopen.bmj.com).

If you have any questions on BMJ Open's open peer review process please email info.bmjopen@bmj.com

BMJ Open

Methodological Considerations for Linking Household and Healthcare Provider Data for Estimating Effective Coverage: A Systematic Review

Journal:	*BMJ Open*
Manuscript ID	bmjopen-2020-045704
Article Type:	Original research
Date Submitted by the Author:	13-Oct-2020

Complete List of Authors:	Carter, Emily; Johns Hopkins University Bloomberg School of Public Health, International Health<br>Leslie, Hannah; Harvard TH Chan School of Public Health, Global Health and Population<br>Marchant, Tanya; London Sch Hyg , Disease Control<br>Amouzou, Agbessi; Johns Hopkins University Bloomberg School of Public Health, International Health<br>Munos, Melinda; Johns Hopkins University, Bloomberg School of Public Health, Department of International Health

Keywords:	Public health < INFECTIOUS DISEASES, Quality in health care < HEALTH SERVICES ADMINISTRATION & MANAGEMENT, PUBLIC HEALTH

SCHOLARONE™
Manuscripts

I, the Submitting Author has the right to grant and does grant on behalf of all authors of the Work (as defined in the below author licence), an exclusive licence and/or a non-exclusive licence for contributions from authors who are: i) UK Crown employees; ii) where BMJ has agreed a CC-BY licence shall apply, and/or iii) in accordance with the terms applicable for US Federal Government officers or employees acting as part of their official duties; on a worldwide, perpetual, irrevocable, royalty-free basis to BMJ Publishing Group Ltd ("BMJ") its licensees and where the relevant Journal is co-owned by BMJ to the co-owners of the Journal, to publish the Work in this journal and any other BMJ products and to exploit all rights, as set out in our licence.

The Submitting Author accepts and understands that any supply made under these terms is made by BMJ to the Submitting Author unless you are acting as an employee on behalf of your employer or a postgraduate student of an affiliated institution which is paying any applicable article publishing charge ("APC") for Open Access articles. Where the Submitting Author wishes to make the Work available on an Open Access basis (and intends to pay the relevant APC), the terms of reuse of such Open Access shall be governed by a Creative Commons licence – details of these licences and which Creative Commons licence will apply to this Work are set out in our licence referred to above.

Other than as permitted in any relevant BMJ Author's Self Archiving Policies, I confirm this Work has not been accepted for publication elsewhere, is not being considered for publication elsewhere and does not duplicate material already published. I confirm all authors consent to publication of this Work and authorise the granting of this licence.

For peer review only

Methodological Considerations for Linking Household and Healthcare Provider Data for Estimating Effective Coverage: A Systematic Review

Emily D. Carter*[1], Hannah H. Leslie[2], Tanya Marchant[3], Agbessi Amouzou[1], Melinda Munos[1]

[1] Institute for International Programs, Johns Hopkins Bloomberg School of Public Health, Baltimore, MD, USA

[2] Department of Global Health and Population, Harvard T.H. Chan School of Public Health, Boston, MA, USA

[3] Faculty of Infectious and Tropical Diseases, London School of Hygiene & Tropical Medicine, London, UK

*Corresponding author: Emily Carter, Johns Hopkins Bloomberg School of Public Health, 615 N Wolfe St, Baltimore, MD, 21205, ecarter@jhu.edu

Word Count: 4998

For peer review only - http://bmjopen.bmj.com/site/about/guidelines.xhtml

ABSTRACT

Objective: To assess existing knowledge related to methodological considerations for linking population-based surveys and health facility data to generate effective coverage estimates.

Design: Systematic review of available literature.

Data sources: Medline, Carolina Population Health Center, and Demographic and Health Survey (DHS) publications and hand-search of related or referenced works of all articles included in full text review. The search included publications from 1 January 2000 to 7 June 2019 without language restriction.

Eligibility criteria: Publications explicitly evaluating 1) the suitability of data used in linking analyses, 2) the implications of the design of existing data sources commonly used in linking analyses, and 3) the impact of choice of method for combining datasets to obtain linked coverage estimates.

Results: Of 3192 papers reviewed, 62 publications addressed issues related to linking household and provider datasets. Limited data suggest household surveys can be used to identify sources of care, but their validity in estimating a denominator of intervention need was variable. Methods for collecting provider data and constructing quality indices were variable and presented limitations. There was little empirical data supporting an association between structural, process, and outcome quality. Few studies addressed the influence of the design of common data sources on linking analyses, including imprecise household GIS data, provider sampling frame and sampling design, and estimate stability. There was a lack of concrete evidence around the impact of these factors on linked effective coverage estimates. The most consistent evidence suggested under certain conditions, combining data sets based on geographical proximity (ecological linking) produced similar estimates to linking based on the specific provider utilized (exact-match linking).

Conclusions: Linking household and healthcare provider can leverage existing data sources to generate more informative estimates of intervention coverage and care. However, there is need for additional research to develop evidence-based, standardized best practices for these analyses.

Strengths and limitations of this study

- We systematically reviewed a wide range of methodological issues pertaining to linking population-based and health provider data for effective coverage estimation
- The review was limited by the diversity of terminology and fields related to the linking methodology
- Multiple search strategies were used to minimize the likelihood of overlooking relevant publications
- Results of the review are summarized and related to actionable items and needs for future research

What is already known?

- Linking population-based and provider data is a means of generating effective coverage estimates, however little guidance exists on methodological considerations for linking these data sources

For peer review only - http://bmjopen.bmj.com/site/about/guidelines.xhtml

What are the new findings?

- 62 publications address issues related to the 1) the suitability of data used in linking analyses, 2) the implications of the design of existing data sources commonly used in linking analyses, and 3) the impact of choice of method for combining datasets to obtain linked coverage estimates
- There was variable and limited evidence on the suitability of data household and provider data, particularly collecting and constructing indicators of provider quality, and on the implications of the design of existing data sources
- The most consistent evidence suggested under certain conditions, combining data sets based of geographical proximity or administrative unit produced similar estimates to linking based on the specific provider utilized

What do the new findings imply?

- There is need for additional research to develop evidence-based, standardized best practices for linked analyses of health system and population data

For peer review only

For peer review only - http://bmjopen.bmj.com/site/about/guidelines.xhtml

BACKGROUND

There is growing demand for tracking progress towards the sustainable development goals (SDGs) through effective coverage estimates [1,2]. Effective coverage measures assess not only the proportion of individuals in need of an intervention who receive it, but also the content and quality of services received with an aim to estimate the proportion of individuals receiving the health benefit of an intervention [2]. Numerous publications have estimated effective coverage [3] using a range of methods and measures to define intervention need, receipt, and quality.

Linking household and health provider data is a promising means of generating effective coverage estimates that provide population-based estimates and incorporate data on service quality from health facilities. Data from household surveys can provide a population-based estimate of intervention need and care-seeking for services, such as the proportion of women with a recent live birth who delivered in a health facility. However, a number of maternal, newborn, and child health interventions [4] cannot be accurately measured through household surveys due to reporting errors and biases by respondents (e.g., the proportion of women who received a uterotonic during delivery). Health provider assessments yield information on provider quality, including available infrastructure, commodities, equipment, human resources, and potentially provision of care. Provider data do not capture need for care in the population, care-seeking behavior, or the experience of individuals who do not access the formal health system. Linking these two data sources can provide a more complete picture of population access to and coverage of high-quality health services, for example the proportion of women who delivered at a health facility with sufficient structural resources and competence to provide appropriate labor and delivery care.

There are many approaches for combining household and provider datasets [5]. The results depend on the choice of data and of methods for combining datasets. However, very limited guidance exists to guide decision making. We conducted a systematic review to understand the current evidence base for effective coverage linking methods and identify needs for further research.

METHODS

We searched for papers addressing methods or assumptions regarding: 1) the suitability of household and provider[1] data used in linking analyses, 2) the implications of the design of existing household (DHS and MICS) and provider (SPA and SARA) data sources commonly used in linking analyses, and 3) the impact of choice of method for combining datasets to obtain linked coverage estimates.

Our primary search was conducted in Medline. The search was limited to papers published between January 1, 2000 and June 7, 2019 that included terms related to 1) effective coverage, benchmarking, system dynamics, or universal health coverage metrics, or 2) structural, process, and/or health outcome quality, 3) linking analyses using terms adapted from Do and colleagues [5], 4) validity of self-report health indicators, and 5) spatial methods for measuring utilization or distance to care. A full list of Medline search terms and PRISMA checklist are presented in Supplementary File 1 and 2, respectively. The search was conducted using English-language terms; however, publications in English, Spanish, and French were reviewed if captured in the search. Additionally, we conducted searches using these criteria

[1] We define health providers as health care outlets such as health facilities, pharmacies, and community-based health workers

For peer review only

in Population Health Metrics (which was not fully indexed in Medline at the time of our search), the Carolina Population Health Center, and DHS publications. In a second step, we hand-searched the references of a systematic review by Do and colleagues on linking household and facility data to estimate coverage of reproductive, maternal, newborn, and child health services [5], and a review by Amouzou and colleagues of effective coverage analyses [3]. We also hand-searched the references, citing works, and journal- or database interface-generated related publications of all articles that passed the title and abstract review. Particularly relevant papers published after the formal search date were included in the review if captured through these snowball review mechanisms.

Publications were reviewed for relevant analyses or commentary related to linking methodologies. Articles were included if they explicitly evaluated or compared assumptions used in linking approaches for at least one of the areas defined above. Title and abstract review were conducted simultaneously. Data extraction was completed by the first author (EC) and included the title, author, year of publication, country or countries included in analysis, data source, and specific analyses or findings relevant to linking loosely categorized by topic areas.

Patient and public involvement

As a systematic review, neither patients nor the public were involved in the design, conduct, reporting, or dissemination plans of our research.

RESULTS

The Medline search produced 3055 publications, along with 97 from the Carolina Population Center, 4 from Population Health Metrics, 12 DHS publications, 35 papers included in the review by Amouzou and colleagues and 49 papers included in the review by Do and colleagues meeting the publication date restrictions. After removing duplicates, 3192 publications were included in the title and abstract review and 218 were included in the full text review. Of those papers included in the full text review, 48 publications addressed a methodological concern related to linking household and provider data and were included in the final review. Fourteen additional publications were identified through the snowball review of references and related works (Fig 1 – PRISMA flow diagram). In total 62 publications addressed a methodological concern, including the suitability of household (n=13) and provider data (n=32) for use in linking analyses, concerns related to the design of existing household (n=6) and provider (n=4) data sources, and methods for combining household and facility data (n=13). A list of publications included in the review and a summary of their contributions to the review are provided in Table 1.

-TABLE 1-

Suitability of household and provider data for linking analyses

Suitability of household data needed for linked estimates

In effective coverage linking analyses, household surveys can be used to estimate the population in need of healthcare, as well as care-seeking behavior. Household surveys must produce valid estimates of these parameters and provide care-seeking data that can be linked to provider assessments. This review identified papers discussing issues in defining intervention need (n=8) and care-seeking (n=5) that should guide selection of indicators for linking.

For peer review only - http://bmjopen.bmj.com/site/about/guidelines.xhtml

Intervention need

Estimation of intervention need may require solely population demographics such as age (e.g., for prevention and health promotion interventions) or may require defining specific illnesses or conditions. The latter is more subject to reporting bias [6]. Multiple studies have shown poor association or biases between maternally-reported symptoms and clinical pneumonia [7,8], malaria [9], and diarrhea [10] in children under five. A handful of studies (n=3) showed maternal report of both maternal and newborn birth complications is variable [11–13]. A simulation by Shengelia and colleagues demonstrated the effect of the divergence of true from perceived intervention need on effective coverage estimates. The authors propose estimating the posterior probability of disease based on responses to symptomatic questions using a Bayesian model to measure disease presence on a probabilistic scale [6]. However, there has been no work on how to integrate these adjusted estimates into effective coverage estimates.

Care-seeking behavior

Four studies addressed the accuracy of respondent report on seeking care. Mothers in Zambia and Mozambique were able to accurately report on the type of health provider where they sought sick child [14] and delivery care [15], respectively. However, studies in two countries suggested women cannot report on the type of healthworker who attended to them during labor and delivery and immediate post-natal care [16,17]. Wang and colleagues note that provider categories are not standardized between population surveys and health system assessments, with population surveys often including vague or overly broad categories that do not directly match SPA/SARA categories and require harmonization [18].

Suitability of healthcare quality data needed for linked estimates

Provider assessments present data on service content and quality for effective coverage linking analyses. However, the measurement, construction, and interpretation of provider quality measures are highly variable and may significantly alter effective coverage estimates. This paper does not present an exhaustive review of healthcare quality measures or the association between levels of quality. A comprehensive summary of quality of care concepts and measurement approaches, along with their relative strengths and limitations, was presented by Hanefeld and colleagues [19]. Publications of particular relevance to linking analyses are noted here, with an emphasis on national provider survey data as the most common source of provider data for linking analyses.

Methods used in assessing provider quality

A review by Nickerson and colleagues found significant variability in the data collected and methods used in health facility assessment tools in LMICs [20]. While SPA and SARA data are the most widely used sources of data on health service delivery in LMICs, one paper noted that these surveys focused primarily on structural quality with less data on provision and experience of care [21]. The lack of process quality data is in part related to the reliance on direct observation of clinical care – a time- and resource-intensive method – to collect these data. None of the studies included in the review used HMIS data to generate linked coverage estimates. A desk review by MCSP found that data collected through HMIS was variable across countries, data recorded within registers often was not transmitted through the system, and only a limited number of indicators collected were related to the provision of health services [22].

For peer review only - http://bmjopen.bmj.com/site/about/guidelines.xhtml

Eight publications assessed alternatives to direct observation of clinical care for collecting process quality data. Two studies found variable association between process quality and maternal perceptions of the quality of care received [23,24]. Agreement between observed care and health records or provider report was also variable [25–27]. A review by Hrisos and colleagues found few studies to support use of patient report, provider self-report, or record review as proxy measures of clinical care quality [28]. In the US, vignettes performed better than chart abstraction for estimating quality [29]. Another review found providers were unable to accurately assess their own performance, with the worst accuracy among the least skilled providers [30]. Five other publications used alternative methods for measuring process quality, including use of vignettes [24,31], register review [24,32], most recent delivery interview [33], and an mHealth tool [34], but did not assess their performance against other measurement methods.

Content of provider quality indices

Most linking papers estimating effective coverage included in this review (n=10) characterized provider quality using structural measures of quality, with or without measures of process quality. Various approaches were used to select items for inclusion in these measures. Measures of structural and process quality were derived from either national or international guidance on minimum service availability and required commodities, equipment, infrastructure, training, or actions. Measures used by effective coverage analyses included SPA or SARA structural indicators [31,35,36] and/or clinical observations [36–38], EmOC functions [26,32,39,40], and provider recall of actions during their last delivery [33,39].

Construction of provider quality indices

In addition to the range of variables used in provider measures, there was no consensus on the approach to use to select and combine variables to generate quality indices. The reviewed publications used a variety of approaches to construct indices including weighted indices [38], simple averages across all indicators or domains [31,37,39], and categorization using set thresholds or relative categories [24,35,40]. Seven publications presented data on the performance of different measurement modes and summary approaches. Two studies found the method of selecting and combining quality indicators had little effect on overall effective coverage estimates [41,42]. However, two other studies found inconsistency in the rankings of health facilities when using different index methods [43,44]. Two studies using PCA to create SPA health service indices found the reduced indices explained only a limited amount of the variance across indicators [44,45]. An analysis of SPA data in ten countries found indices empirically-derived through machine learning captured a large proportion of the service readiness data in the full SPA index, however the selected set of indicators varied across countries, and an index generated through expert review captured very little of the data from the full index [46]. Two studies found that few facilities could meet all requirements when applying a threshold, limiting the utility of the approach [40,43].

Performance of provider measures

Despite the common usage of SPA and SARA data-derived structural and process quality measures, the review found limited data explicitly assessing the association of these measures with each other and health outcomes (n=7). Three studies, two incorporating data from multiple countries, found little association between structural quality and process quality [38,47,48]. However, an analysis of SPA data

For peer review only - http://bmjopen.bmj.com/site/about/guidelines.xhtml

from three countries found a small but significant association between ANC facility structural and process quality and suggests structural quality can limit provider performance when basic infrastructure and commodities are unavailable [42]. Akachi and Kruk emphasized the limited number of studies showing process quality associated with health outcomes [49]. One study found a small association between an obstetric quality index and decreased neonatal mortality in Malawi [50] and another found a national UHC "heath service coverage" index correlated strongly with infant mortality rate and life expectancy [51].

Implications of design of existing household and provider data sources commonly used in linking analyses

Issues related to household and cluster location data

The way in which common household surveys, particularly the DHS and MICS, collect and process location data may also impact the validity of some linked estimates. In many household datasets used for linked analyses, the precise location of individual households is often unknown. The DHS collects central point locations for clusters, rather than household locations, and displaces these points in publicly released datasets [52]. MICS often does not collect or make GIS data available [53]. Imprecision around household location may influence the accuracy of estimates generated by linking household and provider data based on geographic proximity.

Data on household location

The effect of using cluster central point locations (centroids) rather than individual household locations in linking analyses was not addressed by any publication identified in this review. However, four studies looked at the effect of using centroids of varying areal units versus household locations in distance analyses. Two studies found using US census tract [54] and zip-code [55] centroid locations produced little difference in measures of facility access compared to household location. A third study showed use of areal unit centroids resulted in misclassification of household access to health-related facilities, especially in less densely populated rural areas [56]. However, in rural Ghana, measures calculated from village centroids identified the same closest facility as measures from compound locations for over 85% of births [57].

Cluster displacement

Displacement of cluster central points might induce additional error in analyses based on geographic proximity. A DHS analytic report found that ignoring DHS displacement in analyses that used distance to a resource as a covariate resulted in increased bias and mean squared error (MSE). However, this will not affect linking by administrative unit because DHS has restricted displacement to within the representative sample administrative unit since 2009 [52]. A simulation analysis in Rwanda reported DHS cluster displacement produced less misclassification in level of access and relative service quality than healthcare provider sampling [58].

Issues related to provider sampling

Typical sampling designs for healthcare provider data also present issues for linking analyses. Both SPAs and SARAs are sampled independently of household surveys, thus, there may be no sampled facilities near household survey clusters [59]. SPA and SARA surveys typically collect data on a sample, rather

For peer review only - http://bmjopen.bmj.com/site/about/guidelines.xhtml

than census, of public, private, and NGO health facilities and exclude non-facility providers, such as pharmacies or community health workers. In most settings, facilities are sampled and analyzed to be representative of all facilities within a managing authority, level, and/or geographic area, and the results of the provider assessment are not intended to represent the population utilizing health services [59]. For provider assessments conducting direct observations of clinical care, the number and type of interactions observed within each health facility is dependent on patient volume and chance.

Provider sampling frame

Two papers assessed the impact of excluding non-facility providers on linked effective coverage estimates. In Zambia and Cote d'Ivoire, CHWs offered a level of care for sick children similar to first-level public facilities. Excluding these providers reduced estimates of effective coverage in Zambia where CHWs were a significant source of skilled care in rural areas estimates [31], but had little effect in Cote d'Ivoire where they were an insignificant source of care [37]. In both studies, exclusion of pharmacies did not alter effective coverage estimates as they were an uncommon source of care, though they offered moderate structural quality [31,37].

Provider sampling design

Two publications addressed the impact of facility survey sampling designs. At the facility level, Skiles and colleagues' analysis demonstrated that sampling facilities, rather than using a census, led to an underestimation of the adequacy of the health service environment and substantial misclassification error in relative service environment for individual clusters [58]. No studies addressed the suitability of SPA or SARA facility sampling strategies for generating stable quality estimates for use in linking analyses at a level below administrative unit used for the sampling approach.

A Measure Evaluation manual emphasized that data on provision of services (collected through observation of client-staff interactions) and experience of care (collected through client exit interviews) are a sub-sample of the overall survey and representative at the level the survey is sampled to be representative – not at the facility level [59]. This paper proposed multiple linked sampling approaches to capture geographically concordant household and provider data for linked analyses. While multiple studies included in this review used a census or sample of providers derived from a household sample, none implemented this approach at a national scale.

Issues related to timing of surveys used in linked coverage estimates

Both care-seeking behavior and provider quality are likely to vary over time, and both household and provider surveys are conducted infrequently in LMICs (~3-5 years). Linked coverage estimates for RMNCH may cover a long timeframe as the reference period for care-seeking in household surveys varies from 2 weeks (sick child care) to 2-5 years (peripartum care). Population movement and quality improvement efforts at facilities further complicate associations with increasing time lags. The implications of linking household and provider indicators of different temporal periods is unclear.

Stability of provider indicators

No paper in this review specifically addressed the effect of provider indicator stability on linked effective coverage estimates. However, three linking papers presented data on the stability of some health facility

For peer review only - http://bmjopen.bmj.com/site/about/guidelines.xhtml

indicators over time. EQUIP studies in Uganda and Tanzania found moderate variability in the availability of some maternal and newborn health commodities and services over a 2-3 year period [39,60,61].

Stability of household indicators

Care-seeking behavior, including overall rates of care and utilization of different sources of care, may also change over time. Analysis of care-seeking for child illness [62] and maternal healthcare [63] in multiple low- and middle-income countries over time showed high inconsistency in trends across countries. However, no identified studies addressed the consequences of this temporal variability within the context of linking analyses.

Impact of choice of method for combining household and provider data

The approach for combining household and provider data can potentially have a significant impact on linked coverage estimates. Methods used to link data, including exact-match and various types of ecological linking, are defined in Table 2. Exact-match linking assigns provider information to individuals in the target population based on their specific source of care. This approach, while potentially subject to the reporting biases described previously, is considered the most precise approach for combining the two data sets in the absence of individual patient health records [5]. Without data on specific source of care, ecological linking approaches are designed to approximate care-seeking behavior or model healthcare access by linking the target population to sources of care based on geographic proximity or administrative catchment area, making assumptions about service access and use.

-TABLE 2-

Comparison of exact-match and ecological linking methods for estimating effective coverage

Three publications explicitly compared effective coverage estimates generated using exact-match and ecological linking methods (Table 3) [31,37,39]. Estimates generated using the exact-match linking approach were considered the gold-standard measure of effective coverage. All three publications found exact-match linked effective coverage estimates were similar to straight-line [31,37], travel time [31,37], 5-km buffer [31], 10-km buffer [37], and administrative unit [31,37,39] geolinked estimates for antenatal [37], labor and delivery [37,39], postnatal [37] and sick child [31,37] care when linking was restricted by the reported provider category (e.g., hospital, health center, community health worker). Distance-restricted linking approaches, such as linking to providers within a 5 km radius, produced inaccurate results if unlinked events were treated as no care [31]. Restriction of geographic linking to only providers within the reported category of care and/or weighting by providers' relative patient volume improved agreement between the exact-match and ecological linking estimates [37,39]. All three studies also used provider data obtained from a census of health facilities, and therefore the findings may not be applicable when household data are linked to a sample survey of health facilities.

-TABLE 3-

Performance of measures of geographic proximity for ecological linking

Eight studies assessed the performance of geographic measures in assigning households or individuals to their reported source of healthcare. Four studies in sub-Saharan Africa compared the predicted source of care based on geographic proximity against the true source of care. They found straight-line and road distance performed similarly [64], high performance of shortest travel time method [65], and

For peer review only - http://bmjopen.bmj.com/site/about/guidelines.xhtml

better performance of straight-line distance compared to road distance [31,37]. In the US, a more sophisticated approach (2-stage and 3-stage floating catchment area) performed better than alternatives methods in assigning households to their source of care [66]. Three studies in sub-Saharan Africa evaluated use of Theissen boundaries, a method of defining catchment boundaries based on the optimal distance between known providers, in assigning households to the catchment of facilities they utilized. The studies found high performance in some settings [67], but poorer performance related to the use of higher-order facilities [68] and influence of public transportation routes [69].

Statistical challenges

Most linking analyses that have generated effective coverage estimates by assigning individuals the quality score of the reported or linked source of care have derived estimates of uncertainty based on household sampling error and ignored any sampling error around provider data. However, two analyses used the Delta method (73) for estimating the variance of effective coverage estimates generated by multiplying service use and readiness [18,39]. These papers did not elaborate on why the method was chosen or compare the estimates to values generated using alternative approaches. No other papers in this analysis proposed or examined methods to derive measures of uncertainty for linked estimates.

DISCUSSION

This review found a limited number of publications that explicitly addressed methodological issues related to linking household and provider datasets. A summary of key findings and needs for further research is presented in table 4 and discussed below.

-TABLE 4-

We identified a number of papers that critically assessed household and provider data needed for linking analyses. The limited existing data on respondent-reported care-seeking suggest respondents can identify sources of care if not individual healthcare worker cadre, but additional validation in various settings and service areas, such as postnatal care, would be informative. Further, it is essential to ensure that categorization of sources of care in household surveys align with the categories used in provider assessments to facilitate linking datasets. The validity of household survey data for estimating populations in need was more variable. While some populations in need can be clearly defined, others, particularly those requiring symptom-derived diagnoses based on respondent report, have demonstrated potential for bias. Additional work is needed to explore alternative methods for identifying populations in need in population-based data collection.

The content and construction of provider quality indices was highly variable across publications, but largely derived from facility surveys and was informed by international guidelines or recommendations. Methods for collecting provider quality have a number of limitations, and no single method perfectly encompasses all aspects of care [71]. The review found a lack of agreement between measures of quality derived through various means of collection. Overall, there was little empirical data supporting association between structural quality and process quality, and measures of quality and appropriate care or good health outcomes, although the number of reviewed studies was very limited. However, as articulated by Nguhiu and colleagues, there is need to consider quality indicators' "intrinsic value as levers for management action" and application to policy decision-making in addition to their ability to capture or predict associated health gain [36]. Additional research is needed in the short term to

For peer review only - http://bmjopen.bmj.com/site/about/guidelines.xhtml

develop and evaluate new quality indices utilizing existing data sources (e.g., facility surveys, HMIS, and medical records) with an aim of identifying a standardized approach for selecting, combining, and interpreting indicators that reflect aspects of provider quality necessary for delivering appropriate, respectful, and effective care. Longer term, substantial effort is needed to strengthen or adapt existing mechanisms or develop alternative methods for collecting provider quality indicators that can produce timely and informative estimates for tracking effective coverage of key interventions.

Few studies addressed the influence of the design of common data sources on linking analyses, including the impact of imprecise household GIS data, provider sampling frame and sampling design, and estimate stability. However, there was a lack of concrete evidence around the impact of these factors on linked effective coverage estimates. Explicitly evaluating the impact of imprecise household location, sampling design, and temporal gaps between measures within the context of effective coverage estimation would be informative. Mixed results on the inclusion of non-facility providers in provider assessments for effective coverage estimation emphasize the need to empirically assess the utilization and service quality of non-facility providers in a given setting prior to conducting a linking analysis. Although data related to impact on effective coverage estimation were limited, small samples of client-staff observations, sampling of facilities, and temporal gaps between household and provider data have the potential to bias estimates. The available data suggest that developing and testing alternative means of sampling health providers could improve the validity of linked estimates of effective coverage, including evaluating joint sampling approaches proposed by Measure Evaluation [59] or used by other data collection mechanisms such as PMA2020 and the India District Level Household and Facility Survey. No papers effectively addressed statistical concerns in combining household and provider data sources. There is a need to develop and test approaches for estimating uncertainty around linked effective coverage estimates.

The most consistent evidence found through the review was around methods for combining data sets. Three papers compared ecological and exact match linking and reported that ecological linking produced similar estimates to exact-match linking under certain conditions. The agreement between the three publications that compared exact-match and ecological linking is promising. Exact-match linking is considered the most precise method for generating linked estimates; however, ecological linking is often more feasible because it does not require information on exact source of care or data on all providers. The papers further point to the need to maintain data on type of provider from which care was sought or the relative volume of patients seen by providers in order to generate valid estimates of effective coverage. All three studies were conducted in rural sub-Saharan Africa in settings with high utilization of public sector health facilities; additional studies evaluating the performance of these methods in settings with a more diverse healthcare landscape would be informative. Other papers evaluated ecological linking approaches and found similar estimates of access to care or effective coverage using different approaches for assessing geographic proximity, although the ability of methods to capture true source of care was more variable. External to this review, additional data suggests individuals may not always utilize the closest source of care and may bypass providers in favor of providers offering better care [35,72]. These findings along with the analyses comparing exact-match and ecological linking approaches emphasize the need to carefully select methods for performing ecological linking and to control for true care-seeking behavior as much as possible by accounting for the type of provider from which care was sought or weighting by utilization in linking analyses.

For peer review only - http://bmjopen.bmj.com/site/about/guidelines.xhtml

Evidence across the review demonstrates the need for careful choice of methods, data sources, and indicators when conducting studies or analyses to link household and provider data for effective coverage estimation. An exploration of the precise effect of setting characteristics, such as variation in provider quality, on effective coverage estimates is needed to guide decision-making in the selection of linking methods. Once more of these issues have been evaluated, additional tools and guidance to facilitate use of these methods will be needed.

The review was limited by the diversity of terminology and fields related to the linking methodology. However, the use of multiple search strategies minimized likelihood of overlooking relevant publications. Additionally, the diversity of fields, approaches, and questions made it difficult to summarize the findings neatly, emphasizing the need for communication between researchers, more standard terminology, and, ideally, a cohesive research strategy going forward.

CONCLUSIONS

Linking household and healthcare provider data is a promising approach that leverages existing data sources to generate more informative estimates of intervention coverage and care. These methods can potentially address limitations of both household and provider surveys to generate population-based estimates that reflect not only use of services, but also the content and quality of care received and the potential for health benefit. However, there is need for additional research to develop evidence-based, standardized best practices for these analyses. The most pressing priorities identified in this review are: 1) for those collecting data from health systems to explore methods to strengthen existing provider data collection mechanisms and promote temporal and geographic alignment with population-based measures, 2) for those collecting population-based data to address validity of self-reported intervention need and ensure indicators of access and utilization of care are measured to facilitate linking analyses, and 3) for those conducting linked analyses to standardize approaches for generating and interpreting effective coverage indicators, including sources of uncertainty, to ensure we are producing evidence that is harmonized, informative, and actionable for governments and valid for monitoring population health globally.

For peer review only - http://bmjopen.bmj.com/site/about/guidelines.xhtml

For peer review only

Author contributions: Conceived of the study design: EC MM. ICMJE criteria for authorship read and met: EC HL TM AA MM. Agree with manuscript and conclusions: EC HL TM AA MM. Conducted review: EC. Drafted paper: EC. All authors read, edited, and approved the manuscript.

Competing Interests: We declare no competing interests in accordance with ICMJE Conflict of Interest Guidelines.

Funding: This work was supported by the Improving Coverage Measurement grant (OPP1172551) from the Bill & Melinda Gates Foundation. The funders did not have any role in the design of the study and collection, analysis, and interpretation of data or in writing the manuscript.

Data Sharing: As a review article, this article reports data from previously published studies.

Ethics Approval: Ethical approval was not required as this review only included publicly available, published data.

Patient and public involvement Patients and/or the public were not involved in the design, or conduct, or reporting, or dissemination plans of this research.

Patient consent for publication Not required.

Provenance and peer review Not commissioned; externally peer reviewed.

For peer review only

REFERENCES

1 Ng M, Fullman N, Dieleman JL, *et al.* Effective coverage: a metric for monitoring Universal Health Coverage. *PLoS Med* 2014;**11**:e1001730. doi:10.1371/journal.pmed.1001730

2 Marsh AD, Muzigaba M, Diaz T, *et al.* Effective coverage measurement in maternal, newborn, child, and adolescent health and nutrition: progress, future prospects, and implications for quality health systems. *Lancet Glob Health* 2020;**8**:e730–6. doi:10.1016/S2214-109X(20)30104-2

3 Amouzou A, Leslie HH, Ram M, *et al.* Advances in the measurement of coverage for RMNCH and nutrition: from contact to effective coverage. *BMJ Global Health* 2019;**e001297**.

4 Bryce J, Arnold F, Blanc A, *et al.* Measuring Coverage in MNCH: New Findings, New Strategies, and Recommendations for Action. *PLOS Med* 2013;**10**:e1001423. doi:10.1371/journal.pmed.1001423

5 Do M, Micah A, Brondi L, *et al.* Linking household and facility data for better coverage measures in reproductive, maternal, newborn, and child health care: systematic review. *J Glob Health* 2016;**6**:020501. doi:10.7189/jogh.06.020501

6 Shengelia B, Tandon A, Adams OB, *et al.* Access, utilization, quality, and effective coverage: an integrated conceptual framework and measurement strategy. *Soc Sci Med 1982* 2005;**61**:97–109. doi:10.1016/j.socscimed.2004.11.055

7 Ayede AI, Kirolos A, Fowobaje KR, *et al.* A prospective validation study in South-West Nigeria on caregiver report of childhood pneumonia and antibiotic treatment using Demographic and Health Survey (DHS) and Multiple Indicator Cluster Survey (MICS) questions. *J Glob Health* 2018;**8**:020806. doi:10.7189/jogh.08-020806

8 Hazir T, Begum K, El Arifeen S, *et al.* Measuring coverage in MNCH: a prospective validation study in Pakistan and Bangladesh on measuring correct treatment of childhood pneumonia. *PLoS Med* 2013;**10**:e1001422. doi:10.1371/journal.pmed.1001422

9 D'Acremont V, Lengeler C, Genton B. Reduction in the proportion of fevers associated with Plasmodium falciparum parasitaemia in Africa: a systematic review. *Malar J* 2010;**9**:240. doi:10.1186/1475-2875-9-240

10 Walker CLF, Fontaine O, Black RE. Measuring Coverage in MNCH: Current Indicators for Measuring Coverage of Diarrhea Treatment Interventions and Opportunities for Improvement. *PLOS Med* 2013;**10**:e1001385. doi:10.1371/journal.pmed.1001385

11 Keenan K, Hipwell A, McAloon R, *et al.* Concordance between maternal recall of birth complications and data from obstetrical records. *Early Hum Dev* 2017;**105**:11–5. doi:10.1016/j.earlhumdev.2017.01.003

12 Zimmerman LA, Shiferaw S, Seme A, *et al.* Evaluating consistency of recall of maternal and newborn care complications and intervention coverage using PMA panel data in SNNPR, Ethiopia. *PLoS ONE* 2019;**14**. doi:10.1371/journal.pone.0216612

For peer review only - http://bmjopen.bmj.com/site/about/guidelines.xhtml

13 Chang KT, Mullany LC, Khatry SK, *et al.* Validation of maternal reports for low birthweight and preterm birth indicators in rural Nepal. *J Glob Health* 2018;**8**.

14 Carter ED, Ndhlovu M, Munos M, *et al.* Validity of maternal report of care-seeking for childhood illness. *J Glob Health* 2018;**8**:010602. doi:10.7189/jogh.08.010602

15 Stanton CK, Rawlins B, Drake M, *et al.* Measuring Coverage in MNCH: Testing the Validity of Women's Self-Report of Key Maternal and Newborn Health Interventions during the Peripartum Period in Mozambique. *PLOS ONE* 2013;**8**:e60694. doi:10.1371/journal.pone.0060694

16 Blanc AK, Diaz C, McCarthy KJ, *et al.* Measuring progress in maternal and newborn health care in Mexico: validating indicators of health system contact and quality of care. *BMC Pregnancy Childbirth* 2016;**16**:255. doi:10.1186/s12884-016-1047-0

17 Blanc AK, Warren C, McCarthy KJ, *et al.* Assessing the validity of indicators of the quality of maternal and newborn health care in Kenya. *J Glob Health* 2016;**6**:010405. doi:10.7189/jogh.06.010405

18 Wang W, Mallick L, Allen C, *et al.* Effective coverage of facility delivery in Bangladesh, Haiti, Malawi, Nepal, Senegal, and Tanzania. Published Online First: 1 August 2018.https://dhsprogram.com/publications/publication-as65-analytical-studies.cfm (accessed 17 May 2019).

19 Hanefeld J, Powell-Jackson T, Balabanova D. Understanding and measuring quality of care: dealing with complexity. *Bull World Health Organ* 2017;**95**:368.

20 Nickerson JW, Adams O, Attaran A, *et al.* Monitoring the ability to deliver care in low- and middle-income countries: a systematic review of health facility assessment tools. *Health Policy Plan* 2015;**30**:675–86. doi:10.1093/heapol/czu043

21 Sheffel A, Karp C, Creanga AA. Use of Service Provision Assessments and Service Availability and Readiness Assessments for monitoring quality of maternal and newborn health services in low-income and middle-income countries. *BMJ Glob Health* 2018;**3**. doi:10.1136/bmjgh-2018-001011

22 Maternal and Child Survival Program (MCSP). What Data on Maternal and Newborn Health Do National Health Management Information Systems Include? 2018. https://www.mcsprogram.org/resource/what-data-on-maternal-and-newborn-health-do-national-health-management-information-systems-include/ (accessed 20 May 2019).

23 Diamond-Smith N, Sudhinaraset M, Montagu D. Clinical and perceived quality of care for maternal, neonatal and antenatal care in Kenya and Namibia: the service provision assessment. *Reprod Health* 2016;**13**:92. doi:10.1186/s12978-016-0208-y

24 Larson E, Hermosilla S, Kimweri A, *et al.* Determinants of perceived quality of obstetric care in rural Tanzania: a cross-sectional study. *BMC Health Serv Res* 2014;**14**:483. doi:10.1186/1472-6963-14-483

25 Hoogenboom G, Thwin MM, Velink K, *et al.* Quality of intrapartum care by skilled birth attendants in a refugee clinic on the Thai-Myanmar border: a survey using WHO Safe Motherhood Needs Assessment. *BMC Pregnancy Childbirth* 2015;**15**:17. doi:10.1186/s12884-015-0444-0

For peer review only - http://bmjopen.bmj.com/site/about/guidelines.xhtml

26 Nesbitt RC, Lohela TJ, Manu A, *et al.* Quality along the Continuum: A Health Facility Assessment of Intrapartum and Postnatal Care in Ghana. *PLOS ONE* 2013;**8**:e81089. doi:10.1371/journal.pone.0081089

27 Osen H, Chang D, Choo S, *et al.* Validation of the World Health Organization tool for situational analysis to assess emergency and essential surgical care at district hospitals in Ghana. *World J Surg* 2011;**35**:500–4. doi:10.1007/s00268-010-0918-1

28 Hrisos S, Eccles MP, Francis JJ, *et al.* Are there valid proxy measures of clinical behaviour? a systematic review. *Implement Sci* 2009;**4**:37. doi:10.1186/1748-5908-4-37

29 Peabody JW, Luck J, Glassman P, *et al.* Comparison of Vignettes, Standardized Patients, and Chart Abstraction: A Prospective Validation Study of 3 Methods for Measuring Quality. *JAMA* 2000;**283**:1715–22. doi:10.1001/jama.283.13.1715

30 Davis DA, Mazmanian PE, Fordis M, *et al.* Accuracy of Physician Self-assessment Compared With Observed Measures of Competence: A Systematic Review. *JAMA* 2006;**296**:1094–102. doi:10.1001/jama.296.9.1094

31 Carter ED, Ndhlovu M, Eisele TP, *et al.* Evaluation of methods for linking household and health care provider data to estimate effective coverage of management of child illness: results of a pilot study in Southern Province, Zambia. *J Glob Health* 2018;**8**:010607. doi:10.7189/jogh.08.010607

32 Wilunda C, Oyerinde K, Putoto G, *et al.* Availability, utilisation and quality of maternal and neonatal health care services in Karamoja region, Uganda: a health facility-based survey. *Reprod Health* 2015;**12**:30. doi:10.1186/s12978-015-0018-7

33 Marchant T, Tilley-Gyado RD, Tessema T, *et al.* Adding Content to Contacts: Measurement of High Quality Contacts for Maternal and Newborn Health in Ethiopia, North East Nigeria, and Uttar Pradesh, India. *PLOS ONE* 2015;**10**:e0126840. doi:10.1371/journal.pone.0126840

34 Mboya D, Mshana C, Kessy F, *et al.* Embedding systematic quality assessments in supportive supervision at primary healthcare level: application of an electronic Tool to Improve Quality of Healthcare in Tanzania. *BMC Health Serv Res* 2016;**16**:578. doi:10.1186/s12913-016-1809-4

35 Kanyangarara M, Munos MK, Walker N. Quality of antenatal care service provision in health facilities across sub-Saharan Africa: Evidence from nationally representative health facility assessments. *J Glob Health* 2017;**7**:021101. doi:10.7189/jogh.07.021101

36 Nguhiu PK, Barasa EW, Chuma J. Determining the effective coverage of maternal and child health services in Kenya, using demographic and health survey data sets: tracking progress towards universal health coverage. *Trop Med Int Health* 2017;**22**:442–53. doi:10.1111/tmi.12841

37 Munos M, Maiga A, Do M, *et al.* Linking household survey and health facility data for effective coverage measures: a comparison of ecological and individual linking methods using the Multiple Indicator Cluster Survey in Côte d'Ivoire. *J Glob Health* 2018;**8**.

For peer review only

For peer review only - http://bmjopen.bmj.com/site/about/guidelines.xhtml

38 Leslie HH, Sun Z, Kruk ME. Association between infrastructure and observed quality of care in 4 healthcare services: A cross-sectional study of 4,300 facilities in 8 countries. *PLoS Med* 2017;**14**:e1002464. doi:10.1371/journal.pmed.1002464

39 Willey B, Waiswa P, Kajjo D, *et al.* Linking data sources for measurement of effective coverage in maternal and newborn health: what do we learn from individual- vs ecological-linking methods? *J Glob Health* 2018;**8**:010601. doi:10.7189/jogh.08.010601

40 Gabrysch S, Simushi V, Campbell OMR. Availability and distribution of, and geographic access to emergency obstetric care in Zambia. *Int J Gynecol Obstet* 2011;**114**:174–9. doi:10.1016/j.ijgo.2011.05.007

41 Lozano R, Soliz P, Gakidou E, *et al.* Benchmarking of performance of Mexican states with effective coverage. *Lancet Lond Engl* 2006;**368**:1729–41. doi:10.1016/S0140-6736(06)69566-4

42 Sheffel A. Association between Antenatal Care Facility Readiness and Provision of Care at the Individual and Facility Level in Haiti, Malawi, and Tanzania. 2018.

43 Fisseha G, Berhane Y, Worku A, *et al.* Quality of the delivery services in health facilities in Northern Ethiopia. *BMC Health Serv Res* 2017;**17**:187. doi:10.1186/s12913-017-2125-3

44 Mallick L, Wang W, Temsah G. A comparison of summary measures of quality of service and quality of care for family planning in Haiti, Malawi, and Tanzania. Published Online First: 1 August 2017.https://dhsprogram.com/publications/publication-mr20-methodological-reports.cfm (accessed 17 May 2019).

45 Jackson EF, Siddiqui A, Gutierrez H, *et al.* Estimation of indices of health service readiness with a principal component analysis of the Tanzania Service Provision Assessment Survey. *BMC Health Serv Res* 2015;**15**:536. doi:10.1186/s12913-015-1203-7

46 Leslie HH, Zhou X, Spiegelman D, *et al.* Health system measurement: Harnessing machine learning to advance global health. *PLOS ONE* 2018;**13**:e0204958. doi:10.1371/journal.pone.0204958

47 Kruk ME, Chukwuma A, Mbaruku G, *et al.* Variation in quality of primary-care services in Kenya, Malawi, Namibia, Rwanda, Senegal, Uganda and the United Republic of Tanzania. *Bull World Health Organ* 2017;**95**:408–18. doi:10.2471/BLT.16.175869

48 Zurovac D, Guintran J-O, Donald W, *et al.* Health systems readiness and management of febrile outpatients under low malaria transmission in Vanuatu. *Malar J* 2015;**14**:489. doi:10.1186/s12936-015-1017-4

49 Akachi Y, Kruk ME. Quality of care: measuring a neglected driver of improved health. *Bull World Health Organ* 2017;**95**:465–72. doi:10.2471/BLT.16.180190

50 Leslie HH, Fink G, Nsona H, *et al.* Obstetric Facility Quality and Newborn Mortality in Malawi: A Cross-Sectional Study. *PLoS Med* 2016;**13**:e1002151. doi:10.1371/journal.pmed.1002151

For peer review only

For peer review only - http://bmjopen.bmj.com/site/about/guidelines.xhtml

51 Leegwater A, Wong W, Avila C. A concise, health service coverage index for monitoring progress towards universal health coverage. *BMC Health Serv Res* 2015;**15**:230. doi:10.1186/s12913-015-0859-3

52 Perez-Haydrich C, Warren JL, Burgert CR, *et al.* Guidelines on the use of DHS GPS data. Published Online First: 2013.https://dhsprogram.com/publications/publication-SAR8-Spatial-Analysis-Reports.cfm (accessed 14 Sep 2018).

53 FAQ - UNICEF MICS. http://mics.unicef.org/faq (accessed 19 Jun 2019).

54 Bliss RL, Katz JN, Wright EA, *et al.* Estimating Proximity to Care: Are straight line and zipcode centroid distances acceptable proxy measures? *Med Care* 2012;**50**:99–106. doi:10.1097/MLR.0b013e31822944d1

55 Jones SG, Ashby AJ, Momin SR, *et al.* Spatial implications associated with using Euclidean distance measurements and geographic centroid imputation in health care research. *Health Serv Res* 2010;**45**:316–327.

56 Healy MA, Gilliland JA. Quantifying the magnitude of environmental exposure misclassification when using imprecise address proxies in public health research. *Spat Spatio-Temporal Epidemiol* 2012;**3**:55–67. doi:10.1016/j.sste.2012.02.006

57 Nesbitt RC, Gabrysch S, Laub A, *et al.* Methods to measure potential spatial access to delivery care in low- and middle-income countries: a case study in rural Ghana. *Int J Health Geogr* 2014;**13**:25. doi:10.1186/1476-072X-13-25

58 Skiles MP, Burgert CR, Curtis SL, *et al.* Geographically linking population and facility surveys: methodological considerations. *Popul Health Metr* 2013;**11**:14. doi:10.1186/1478-7954-11-14

59 Turner A, Angeles G, Tsui A, *et al.* Sampling Manual for Facility Surveys for Population, Maternal Health, Child Health and STD Programs in Developing Countries, July 2001 — MEASURE Evaluation Manual Series, No. 3. 2001.https://www.measureevaluation.org/resources/publications/ms-01-03 (accessed 17 May 2019).

60 Baker U, Peterson S, Marchant T, *et al.* Identifying implementation bottlenecks for maternal and newborn health interventions in rural districts of the United Republic of Tanzania. *Bull World Health Organ* 2015;**93**:380–9. doi:10.2471/BLT.14.141879

61 Marchant T, Nathan R, Jones C, *et al.* Individual, facility and policy level influences on national coverage estimates for intermittent preventive treatment of malaria in pregnancy in Tanzania. *Malar J* 2008;**7**:260. doi:10.1186/1475-2875-7-260

62 Winter R, Wang W, Florey L, *et al. Levels and Trends in Care Seeking for Childhood Illness in USAID MCH Priority Countries. DHS Comparative Reports No. 38.* Rockville, MD, USA: : ICF International 2015.

63 Wang W, Soumya A, Wang S, *et al. Levels and Trends in the Use of Maternal Health Services in Developing Countries. DHS Comparative Reports No. 26.* Calverton, MD, USA: : ICF Macro 2011.

For peer review only - http://bmjopen.bmj.com/site/about/guidelines.xhtml

64 Noor AM, Amin AA, Gething PW, *et al.* Modelling distances travelled to government health services in Kenya. *Trop Med Int Health TM IH* 2006;**11**:188–96. doi:10.1111/j.1365-3156.2005.01555.x

65 Tanser F, Gijsbertsen B, Herbst K. Modelling and understanding primary health care accessibility and utilization in rural South Africa: An exploration using a geographical information system. *Soc Sci Med* 2006;**63**:691–705. doi:10.1016/j.socscimed.2006.01.015

66 Delamater PL, Shortridge AM, Kilcoyne RC. Using floating catchment area (FCA) metrics to predict health care utilization patterns. *BMC Health Serv Res* 2019;**19**:144. doi:10.1186/s12913-019-3969-5

67 Tsoka JM, Le Sueur D. Using GIS to measure geographical accessibility to primary health care in rural South Africa: research in action. *South Afr J Sci* 2004;**100**:329–30.

68 Gething PW, Noor AM, Zurovac D, *et al.* Empirical modelling of government health service use by children with fevers in Kenya. *Acta Trop* 2004;**91**:227–37. doi:10.1016/j.actatropica.2004.05.002

69 Tanser F, Hosegood V, Benzler J, *et al.* New approaches to spatially analyse primary health care usage patterns in rural South Africa. *Trop Med Int Health* 2001;**6**:826–38. doi:10.1046/j.1365-3156.2001.00794.x

70 Oehlert GW. A Note on the Delta Method. *Am Stat* 1992;**46**:27–9. doi:10.1080/00031305.1992.10475842

71 Brook RH, McGlynn EA, Cleary PD. Measuring Quality of Care. *N Engl J Med* 1996;**335**:966–70. doi:10.1056/NEJM199609263351311

72 Akin JS, Hutchinson P. Health-care facility choice and the phenomenon of bypassing. *Health Policy Plan* 1999;**14**:135–51.

For peer review only

For peer review only - http://bmjopen.bmj.com/site/about/guidelines.xhtml

Table 1. Summary of publications included in the review and contribution to the literature

AUTHOR	YEAR	COUNTRY	KEY METHOD CONTRIBUTION
**SUITABILITY OF HOUSEHOLD AND PROVIDER DATA FOR LINKING ANALYSES** • **Household data needed for linked estimates**			
Ayede **[7]**	2018	Nigeria	• Accuracy of maternal report of pneumonia symptoms measured through household survey
Blanc **[16]**	2016	Mexico	• Accuracy of maternal report of delivery / immediate PNC attendant measured through household survey
Blanc **[17]**	2016	Kenya	• Accuracy of maternal report of delivery / immediate PNC attendant measured through household survey
Carter **[14]**	2018	Zambia	• Accuracy of maternal report of care-seeking for child illness measured through household survey
Chang **[13]**	2018	Nepal	• Accuracy of maternal report of birthweight and preterm birth measured through household survey
D'Acremont **[9]**	2010	SSA	• Reduced proportion of fever cases that are malaria
Hazir **[8]**	2013	Pakistan & Bangladesh	• Accuracy of maternal report of pneumonia symptoms measured through household survey
Keenan **[11]**	2017	US	• Accuracy of maternal recall of birth complications
Shengelia **[6]**	2005	-	• Effect of true versus perceived intervention need on effective coverage estimation
Stanton **[15]**	2013	Mozambique	• Accuracy of maternal report of place of delivery measured through household surveys
Walker **[10]**	2013	-	• Issues with measurement of child diarrhea through household surveys
Wang **[18]**	2018	Multiple Regions	• Issues with provider categories and alignment between DHS and SPA surveys
Zimmerman **[12]**	2019	Ethiopia	• Reliability of maternal report of maternal and newborn birth complications
**SUITABILITY OF HOUSEHOLD AND PROVIDER DATA FOR LINKING ANALYSES** • **Provider data needed for linked estimates**			
Akachi **[49]**	2017	-	• Need for global benchmarks for quality • Lack on data linking quality with health outcomes
Carter **[31]**	2018	Zambia	• Quality score for child health effective coverage
Davis **[30]**	2006	High-income countries	• Agreement between provider self-assessment and observed quality
Diamond-Smith **[23]**	2016	Kenya & Namibia	• Association between maternal perception of care and measured structural and process quality
Fisseha **[43]**	2017	Ethiopia	• Internal consistency of structural and process quality indicator
Gabrysch **[40]**	2011	Zambia	• Quality score for labor and delivery effective coverage

For peer review only - http://bmjopen.bmj.com/site/about/guidelines.xhtml

Hoogenboom [25]	2015	Thai-Myanmar Border	• Agreement between facility records and observed care
Hrisos [28]	2009	High-income countries	• Systematic review of agreement between observed quality of care and provider self-report, patient-report, and/or chart review
Jackson [45]	2015	Tanzania	• PCA to reduce quality index
Kanyangarara [35]	2017	SSA	• Quality score for ANC effective coverage
Kruk [47]	2017	SSA	• Association between structural and process quality
Larson [24]	2014	Tanzania	• Association between maternal perception of care and service availability and respect<br>• Vignettes for measuring quality
Leegwater [51]	2015	-	• Association between UHC index and infant mortality and life expectancy at national level
Leslie [50]	2016	Malawi	• Association between quality of delivery care and neonatal mortality
Leslie [38]	2017	SSA	• Quality score for ANC, labor and delivery, sick child, and family planning effective coverage<br>• Association between structural and process quality
Leslie [46]	2018	Multiple Regions	• Performance of approaches for generating service readiness indices
Lozano [41]	2006	Mexico	• UHC index using weighted vs simple average of indicators
Mallick [44]	2017	Haiti, Malawi & Tanzania	• Comparison of measures of family planning quality
Marchant [33]	2015	Ethiopia, Nigeria, & India	• Measurement of quality using "last delivery module"
Mboya [34]	2016	Tanzania	• mHealth tool to measure quality
MCSP [22]	2018	Multiple Regions	• Availability and quality of data captured through HMIS
Munos [37]	2018	Cote D'Ivoire	• Quality score for ANC, labor and delivery, PNC, and child health effective coverage
Nesbitt [26]	2013	Ghana	• Quality score for labor and delivery and PNC effective coverage
Nguhiu [36]	2017	Kenya	• Quality score for ANC, labor and delivery, sick child, and family planning effective coverage
Nickerson [20]	2015	Multiple Regions	• Comparison of data collected through health facility assessments
Osen [27]	2011	Ghana	• Agreement between provider reported and observed surgical service quality

For peer review only - http://bmjopen.bmj.com/site/about/guidelines.xhtml

Peabody **[29]**	2000	US	• Agreement between vignettes, chart abstraction, and simulated client measures
Sheffel **[21]**	2018	Multiple Regions	• Summary of quality data collected through SPA and SPA
Sheffel **[42]**	2018	Haiti, Malawi, Tanzania	• Association between structural and process quality
Willey **[39]**	2018	Uganda	• Quality score for labor and delivery and newborn care effective coverage
Wilunda **[32]**	2015	Uganda	• Quality score for maternal and neonatal care effective coverage
Zurovac **[48]**	2015	Vanuatu	• Poor association between structural quality and clinical care in fever management
**IMPLICATIONS OF DESIGN OF EXISTING HOUSEHOLD AND PROVIDER DATA SOURCES COMMONLY USED IN LINKING ANALYSES** • **Household data**			
Bliss **[54]**	2012	USA	• Comparison of distance using centroid versus true location
Healy **[56]**	2012	Canada & UK	• Comparison of distance using centroids of varying areal groupings
Jones **[55]**	2010	US	• Comparison of distance using zip-code centroid versus true household location
Nesbitt **[57]**	2014	Ghana	• Comparison of straight-line distance, network distance, raster and network-based travel time distance measures using village versus compound centroid
Perez-Heydrch **[52]**	2013	-	• Effect of DHS cluster displacement on distance measures
Skiles **[58]**	2013	Rwanda	• Effect of DHS cluster displacement on estimates of service environment
**IMPLICATIONS OF DESIGN OF EXISTING HOUSEHOLD AND PROVIDER DATA SOURCES COMMONLY USED IN LINKING ANALYSES** • **Provider data**			
Carter **[31]**	2018	Zambia	• Effect of excluding non-facility providers from sampling frame on effective coverage estimates
Munos **[37]**	2018	Cote d'Ivoire	• Effect of excluding non-facility providers from sampling frame on effective coverage estimates
Skiles **[58]**	2013	Rwanda	• Effect of facility sampling on estimates of service environment
Turner **[59]**	2001	-	• Limitations of SPA sampling design<br>• Approach for joint sampling of households and facilities for linking analyses
**IMPLICATIONS OF DESIGN OF EXISTING HOUSEHOLD AND PROVIDER DATA SOURCES COMMONLY USED IN LINKING ANALYSES**			

For peer review only - http://bmjopen.bmj.com/site/about/guidelines.xhtml

• **Survey timing**			
Baker **[60]**	2005	Uganda & Tanzania	• Stability of facility diagnostic capacity over time
Marchant **[61]**	2008	Tanzania	• Stability of IPTp stocks
Wang **[63]**	2011	Multiple Regions	• Stability of maternal health care-seeking behaviors measured through household survey over time
Willey **[39]**	2018	Uganda	• Stability of facility infrastructure indicators for labor, delivery, and newborn care
Winter **[62]**	2015	Multiple Regions	• Stability of care-seeking for child illness behaviors measured through household survey over time
IMPACT OF CHOICE OF METHOD FOR COMBINING HOUSEHOLD AND PROVIDER DATA<br>• **Comparison of exact-match and ecological linking methods for estimating effective coverage**			
Carter **[31]**	2018	Zambia	• Comparison of exact-match and ecological linking methods in estimating effective coverage in sick child care
Munos **[37]**	2018	Cote d'Ivoire	• Comparison of exact-match and ecological linking methods in estimating effective coverage in ANC, labor and delivery, PNC, and sick child care
Willey **[39]**	2017	Uganda	• Comparison of exact-match and ecological linking methods in estimating effective coverage in ANC, labor and delivery, PNC, and sick child care
IMPACT OF CHOICE OF METHOD FOR COMBINING HOUSEHOLD AND PROVIDER DATA<br>• **Performance of measures of geographic proximity for ecological linking**			
Carter **[31]**	2018	Zambia	• Comparison of true-source of care for child illness to straight-line and road distance measures
Delamater **[66]**	2019	US	• Comparison of FCA, simple distance, and Huff distance measure against true utilization patterns
Gething **[68]**	2004	Kenya	• Comparison of Theissen boundaries and true utilization patterns
Munos **[37]**	2018	Cote d'Ivoire	• Comparison of true-source of care for ANC, labor and delivery, PNC, and child illness to straight-line and road distance measures
Noor **[64]**	2006	Kenya	• Comparison of true-source of care for child fever to closest by Euclidian and road distance
Tanser **[69]**	2001	South Africa	• Comparison of Theissen boundaries and true utilization patterns
Tanser **[65]**	2006	South Africa	• Comparison of typical source of care to closest by travel time
Tsoka **[67]**	2004	South Africa	• Comparison of Theissen boundaries and true utilization patterns
IMPACT OF CHOICE OF METHOD FOR COMBINING HOUSEHOLD AND PROVIDER DATA<br>• **Statistical challenges**			

For peer review only - http://bmjopen.bmj.com/site/about/guidelines.xhtml

Wang [18]	2018	Multiple Regions	• Use of Delta method for estimating effective coverage variance
Willey [39]	2018	Uganda	• Use of Delta method for estimating effective coverage variance

For peer review only

For peer review only - http://bmjopen.bmj.com/site/about/guidelines.xhtml

Table 2. Table of linking approaches

APPROACH	METHOD
**EXACT-MATCH**	Link to specific reported source of care
**ECOLOGICAL**	Link to one or more providers based on geographic proximity or administrative association
**Geographic Proximity**	
• **Straight-line /Euclidean distance**	Closest by absolute (crow-flies) distance
• **Manhattan distance**	Closest by sum of horizontal and vertical distance between points on a grid (blockwise)
• **Minokowski distance**	Closest by weighted average of Euclidean and Manhattan distance
• **Road Distance**	Closest by distance along a road (line and joint) network
• **Raster-based travel time**	Closest by travel time between points on a continous grid surface with variable transit speed coefficients in each cell
• **Network-based travel time**	Closest by travel time along a road network accounting for variable speed and road conditions
• **Buffer**	All providers within a defined radius from household
• **Theissen Polygon**	Define catchment boundaries based on optimal distance between known providers
• **Kernel Density Estimation**	Define relative draw of providers over geographic area weighted by quality
• **Interpolated Surface**	Define continuous surface of provider access or quality by smoothing between provider point data
• **Floating Catchment Area**	Define catchments for known providers allowing for cross-border use (catchment overlap) and distance decay
**Administrative**	All providers within administrative unit boundaries

For peer review only - http://bmjopen.bmj.com/site/about/guidelines.xhtml

Table 3. Exact versus ecological linking estimates for select indicators across studies

	Willey Labor & Delivery Structural QoC		Carter Child Health Urban Structural QoC		Carter Child Health Rural Structural QoC		Munos Labor & Delivery Structural QoC		Munos Labor & Delivery Process QoC		Munos Child Health Structural QoC		Munos Child Health Process QoC	
	% (95% CI)	% Diff	% (95% CI)	% Diff	% (95% CI)	% Diff	% (95% CI)	% Diff	% (95% CI)	% Diff	% (95% CI)	% Diff	% (95% CI)	% Diff
**Exact-match**	9.86 (3.2-16.5)	REF	49 (43.6-54.5)	REF	60.3 (55.6-65.1)	REF	37.2 (30.5-43.9	REF	40.1 (32.9-47.3)	REF	22.9 (18.2-27.5)	REF	16.8 (12.8-20.8)	REF
**Ecological - Geographic**														
Absolute Distance							36.5 (29.5-43.5)	-1.9%	39.8 (32.2-47.5)	-0.7%	18.2* (12.3-24.1)	-20.5%	14.3 (9.1-19.6)	-14.9%
Absolute Distance & Provider Category[a]			49.1 (43.7-54.6)	0.2%	61.1 (56.3-65.9)	1.3%	37 (30.0-44.0)	-0.5%	39.6 (32.1-47.1)	-1.2%	20.8* (16.1-25.4)	-9.2%	16.5 (12.2-20.7)	-1.8%
Road Distance							36.8 (30.0-44.0)	-1.1%	40.4 (32.6-48.1)	0.7%	16* (10.9-21.1)	-30.1%	13.8 (8.5-19.1)	-17.9%
Road Distance & Provider Category[a]			48.7 (43.2-54.1)	-0.6%	58.8 (54.1-63.5)	-2.5%	37.5 (30.4-44.6)	0.8%	40.2 (32.5-47.9)	0.2%	20.2* (16.0-24.4)	-11.8%	16.5 (12.3-21.8)	-1.8%
Radius 5 km & Provider Category[a]			49.2 (43.7-54.7)	0.4%	59.4 (54.8-64.1)	-1.5%								
Radius 10 km – Unweighted[b]							35.3* (29.3-41.4)	-5.1%	39.1 (32.0-46.2)	-2.5%	18.8* (14.9-22.7)	-17.9%	15.7 (12.4-19.1)	-6.5%
Radius 10 km – Weighted[c]							37.5 (30.6-44.4)	0.8%	39.8 (32.5-47.1)	-0.7%	19.1* (15.1-23.0)	-16.6%	15.6 (12.1-19.1)	-7.1%
KDE - Single			71.8* (69.3-74.2)	46.5%	55* (50.4-59.6)	-8.8%								
KDE- Aggregate			74.3* (73.2-75.5)	51.6%	54.9* (50.4-59.5)	-9.0%								

For peer review only

For peer review only - http://bmjopen.bmj.com/site/about/guidelines.xhtml

**Ecological - Administrative**														
Facility Catchment & Provider Category[a]			49.1 (43.6-54.6)	0.2%	59.8 (55.1-64.5)	-0.8%								
Sub-District & Provider Category[a]			49.4 (43.9-54.9)	0.8%	57.9 (53.4-62.4)	-4.0%								
District - Unweighted[b]	4.7* (21.4-31.7)	-52.5%					34.9* (29.0-40.8)	-6.2%	39 (32.3-45.7)	-2.7%	17.8* (14.6-21.0)	-22.3%	21* (17.2-24.8)	25.0%
District - Unweighted[b] & Provider Category[a]	11.0 (3.8-18.2)	11.8%					37 (30.4-43.6)	-0.5%	39.7 (32.7-46.7)	-1.0%	20.3* (15.8-24.8)	-11.4%	17.4 (13.3-21.4)	3.6%
District - Weighted[c]							37.9 (31.3-44.4)	1.9%	40.7 (33.7-47.7)	1.5%	19.7* (16.1-23.2)	-14.0%	21.2* (17.4-25.0)	26.2%
District - Weighted[c] & Provider Category[a]							38.8* (31.9-45.7)	4.3%	40.8 (33.6-48.0)	1.7%	21.1* (16.4-25.8)	-7.9%	17.1 (13.1-21.2)	1.8%

[a] Ecological linking restricted to only providers within the category (type of outlet, managing authority, and facility level) reported by survey respondent

[b] Simple average of provider quality scores applied, not accounting for differentials in patient volume

[c] Provider quality scores weighted by provider utilization volume for relevant health area

For peer review only - http://bmjopen.bmj.com/site/about/guidelines.xhtml

Table 4. Summary of evidence related to methodological issues for linking analyses and related needs for future research

**Suitability of household and provider data for linking analyses**<br>• Need valid data on target population for the intervention, and suitable data on service contact/care-seeking<br>• Need provider data reflective of select aspects of quality of care, standardized indices, and clear interpretation of measures		
**Issue**	**Evidence**	**Action**
How valid are data on target population for interventions?	• Symptom/diagnosis-based conditions may be biased<br>• Rare conditions are not captured with sufficient sample	• Explore alternative methods for defining population in need (eg biomarkers, Bayesian modeling of disease probability)
How valid are data on care-seeking?	• Limited data suggest respondent able to identify type of provider but not type of health worker<br>• Inconsistent and sometimes poorly defined provider categories	• Align categories of care across data collection tools<br>• Validate care-seeking in more settings / health areas
How are quality of care data being collected & what are the limitations of these methods?	• Mostly through health facility surveys<br>• HMIS data not widely used – limited QoC data collected<br>• Alternative methods (record review, provider or client report, etc) correlate poorly with provision of services / process quality	• Assess validity of existing QoC measurement methods<br>• Assess availability/usability of HMIS data for EC estimation<br>• Develop and test new methods for assessing provision of care and experience of care
How are quality measures being constructed & what do we know about the performance of these indices?	• Mostly SPA / SARA structural data, limited indicators on provision or experience of care, EmONC signal functions<br>• Variable set of indicators used based on guidelines and standards<br>• Many methods for combining indicators have been tried<br>• Handful of studies comparing methods produced conflicting results	• Develop standardized and validated summary QoC measures
How well do measures of quality track with each other, clinical quality and/or health benefit?	• Limited evidence of weak or no association between 1) structural and process quality, 2) experience of care and provision of care, 3) measured quality and clinical care / health outcomes	• Standardize methods and terminology for defining and interpreting QoC measures to more accurately reflect role in the coverage cascade
**Implications of design of existing household and health provider data sources commonly used in linking analyses**<br>• DHS / MICS household location unknown, cluster location displaced and may introduce imprecision into ecological linking analyses		

For peer review only

For peer review only - http://bmjopen.bmj.com/site/about/guidelines.xhtml

• SPA / SARA often use sample of facilities and subsample of client-staff interactions that may not be representative of true service environment<br>• Household and provider surveys are sampled and conducted independently → data are typically temporally and geographically discordant		
**Issue**	**Evidence**	**Action**
Does imprecise DHS/MICS household location data affect ecological linking results?	• Handful of studies suggest minimal effect on geolinking results	• Assess impact of household versus cluster centroid location versus displaced centroid in ecological linking analyses in multiple settings
How does SPA / SARA sampling design affect estimates?	• 2 studies suggest impact of excluding non-facility providers is context specific<br>• Client-staff interactions sampled to be representative at same level as overall survey – not at facility level<br>• One study showed sampling of facilities resulted in moderate misclassification of service environment across linking methods<br>• Joint sampling method proposed in 2001 – over-sample providers around sampled household clusters	• Assess effect of provider sampling (vs census) on linked estimates<br>• Assess effect of within-facility sampling of healthworkers and client-healthworker observations<br>• Triangulate with other sources of facility data (e.g., HMIS) to take advantage of the greater detail of the SPA assessment with the bigger sample of the facility records<br>• Account for uncertainty in estimates based on the facility-level data (e.g., multilevel structure)<br>• Test alternative sampling methods to improve representativeness of provider survey sampling for clients and healthworkers<br>• Test joint sampling methods for EC estimation
How stable are indicators over time?	• Studies demonstrate moderate indicator variability over months/years<br>• No studies directly related to effect on linking analyses	• Assess stability of key provider and household indicators<br>• Develop and test methods to account for unstable estimates, including more frequent data collection methods (eg, through HMIS) if needed
**Impact of choice of method for combining household and provider data**<br>• Multiple approaches for combining data sets, each with strengths and limitations<br>• Exact-match linking based on specific source of care most precise but ecological linking based on geographic proximity or administrative unit is more feasible		

For peer review only

For peer review only - http://bmjopen.bmj.com/site/about/guidelines.xhtml

Issue	Evidence	Action
How do exact-match and ecological linking approaches compare?	• 3 studies found ecological methods produced estimates similar to exact-match under certain conditions in settings with high use of public providers<br>• Restricting analyses by source of care category and/or weight by utilization volume improved agreement with exact-match	• Assess performance of ecological methods in settings with greater variation in provider landscape, provider quality<br>• Define guidance, such as provider quality variation thresholds, for selection of linking method
How do different ecological linking methods and measures of geographic proximity perform?	• Similar results using straight-line, road distance, and travel time<br>• Variable performance of ecological methods in identifying true source of care / reported category of care	• Identify preferred measures of geographic proximity to use in linking analyses<br>• Create standard, accessible tools for conducting geolinking
What are the statistical challenges in combining data for effective coverage estimation?	• Most analyses base estimates on household sampling error<br>• 2 papers used delta method, but no comparison to other methods	• Develop approaches for estimating uncertainty around linked estimates

For peer review only

Figure 1. PRISMA Flow Diagram

For peer review only

For peer review only - http://bmjopen.bmj.com/site/about/guidelines.xhtml

PRISMA 2009 Flow Diagram

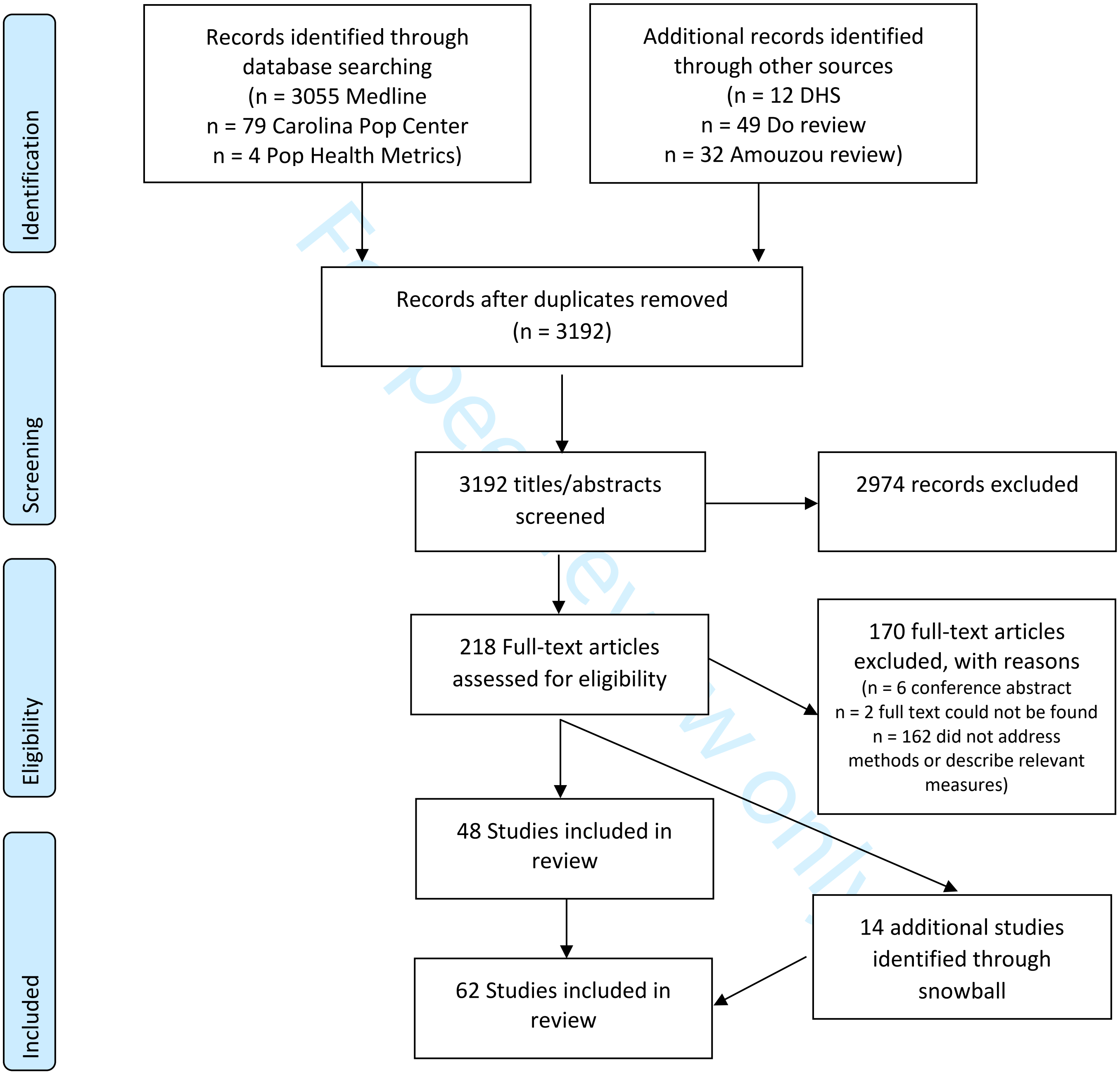

For peer review only - http://bmjopen.bmj.com/site/about/guidelines.xhtml

Supplemental File 1: Medline Search Terms

Term 1: Effective coverage (including related terms e.g. benchmarking, systems dynamics)

Term 2: Linked population-based and health provider analyses

Term 3: Population-based survey validity

Term 4: Health provider quality of care measurement

Term 5: Geospatial measurement of health provider utilization

Term 6: Date and Medline restriction

(Term 1 -OR- Term 2 -OR- Term 3 -OR- Term 4 -OR- Term 5) -AND- Term 6

Term 1:

(((coverage[Title/Abstract]) NOT (insurance[Title/Abstract])) AND (benchmark*[Title/Abstract])) OR (((coverage[Title/Abstract]) NOT (insurance[Title/Abstract])) AND (("systems dynamic framework"[Title/Abstract]) OR ("system dynamics framework"[Title/Abstract]) OR ("systems dynamics framework"[Title/Abstract]))) OR (((“universal health coverage”[Title/Abstract]) OR (UHC[Title/Abstract]) OR (“universal health care”[Title/Abstract])) AND ((coverage[Title/Abstract]) NOT (insurance[Title/Abstract])) AND ((metric*[Title/Abstract]) OR (measur*[Title/Abstract]) OR (score*[Title/Abstract]) OR (indicator*[Title/Abstract]) OR (variable*[Title/Abstract]) OR (index[Title/Abstract]) OR (indices[Title/Abstract]))) OR (("quality-adjusted") AND ((coverage) NOT (insurance[Title/Abstract]))) OR (("effective coverage"[Title/Abstract]) NOT (insurance[Title/Abstract])) OR

Term 2:

((((coverage) NOT (insurance)) AND ((link*[Title/Abstract]) OR (match*[Title/Abstract]) OR (combine*[Title/Abstract]) OR (merge*[Title/Abstract]) OR (attach*[Title/Abstract]) OR (join*[Title/Abstract]) OR (pair*[Title/Abstract]) OR (connect*[Title/Abstract]))) AND (("access to care") OR ("service quality") OR (quality of health care[MeSH Terms]) OR ("service readiness") OR ("service provision") OR ("service delivery") OR ("source of care") OR ("where care was sought") OR ("facility survey") OR ("provider survey") OR ("facility assessment") OR ("provider assessment") OR ("facility data") OR ("provider data") OR ("outlet survey") OR ("outlet assessment") OR ("outlet data")) AND (("service use") OR ("service utilization") OR ("care seeking") OR ("care-seeking") OR ("careseeking") OR ("doctor visit") OR ("clinic visit") OR ("facility visit") OR ("household survey") OR ("household data") OR ("household assessment") OR ("population survey") OR ("demographic survey") OR ("demographic and health survey") OR ("population-based"))) OR

Term 3:

((((("self report"[Title/Abstract]) OR ("self-report"[Title/Abstract]) AND (((maternal[Title/Abstract]) OR (newborn[Title/Abstract]) OR (child[Title/Abstract])) AND (health[Title/Abstract]))) OR ("maternal report"[Title/Abstract]) OR ("maternal recall"[Title/Abstract]) OR ("caregiver recall"[Title/Abstract]) OR ("caregiver report"[Title/Abstract]))) AND ((concordance[Title/Abstract]) OR (accuracy[Title/Abstract]) OR (valid*[Title/Abstract]))) OR

Term 4:

((((“structural quality”[Title/Abstract]) NOT (“structural equation”[Title/Abstract])) OR (infrastructur*[Title/Abstract]) OR (input*[Title/Abstract]) OR (readiness[Title/Abstract]) OR (capacity[Title/Abstract]) OR (“service provision”[Title/Abstract]) OR (“service environment”[Title/Abstract])) AND ((“quality of care”[Title/Abstract]) OR (process[Title/Abstract]) OR (“content of care”[Title/Abstract]) OR (“health outcome”[Title/Abstract]) OR (“health outcomes”[Title/Abstract]) OR ((“population health”[Title/Abstract]) AND (outcome*[Title/Abstract]))) AND ((metric*[Title/Abstract]) OR (measur*[Title/Abstract]) OR (indicator*[Title/Abstract]) OR (score*[Title/Abstract]) OR (variable*[Title/Abstract]) OR (index[Title/Abstract]) OR (indices[Title/Abstract])) AND (survey[Title/Abstract])) OR

Term 5:

((provider[Title/Abstract]) OR (facility[Title/Abstract]) OR (hospital[Title/Abstract]) OR ("source of care"[Title/Abstract])) AND ((geographic[Title/Abstract]) OR (spatial[Title/Abstract]) OR (GIS[Title/Abstract])) AND ((measur*[Title/Abstract]) OR (model*[Title/Abstract])) AND ((utilization[Title/Abstract]) OR ("usage"[Title/Abstract]) OR (distance[Title/Abstract]))) AND

Term 6:

(medline[sb]) AND ("2000/01/01"[PDat] : "2019/06/07"[PDat])

For peer review only

PRISMA 2009 Checklist

Supplemental File 2: PRISMA Checklist

Section/topic	#	Checklist item	Reported on page #
TITLE			
Title	1	Identify the report as a systematic review, meta-analysis, or both.	1
ABSTRACT			
Structured summary	2	Provide a structured summary including, as applicable: background; objectives; data sources; study eligibility criteria, participants, and interventions; study appraisal and synthesis methods; results; limitations; conclusions and implications of key findings; systematic review registration number.	2
INTRODUCTION			
Rationale	3	Describe the rationale for the review in the context of what is already known.	4
Objectives	4	Provide an explicit statement of questions being addressed with reference to participants, interventions, comparisons, outcomes, and study design (PICOS).	4
METHODS			
Protocol and registration	5	Indicate if a review protocol exists, if and where it can be accessed (e.g., Web address), and, if available, provide registration information including registration number.	-
Eligibility criteria	6	Specify study characteristics (e.g., PICOS, length of follow-up) and report characteristics (e.g., years considered, language, publication status) used as criteria for eligibility, giving rationale.	4
Information sources	7	Describe all information sources (e.g., databases with dates of coverage, contact with study authors to identify additional studies) in the search and date last searched.	4-5
Search	8	Present full electronic search strategy for at least one database, including any limits used, such that it could be repeated.	4 (supp. file)
Study selection	9	State the process for selecting studies (i.e., screening, eligibility, included in systematic review, and, if applicable, included in the meta-analysis).	4-5
Data collection process	10	Describe method of data extraction from reports (e.g., piloted forms, independently, in duplicate) and any processes for obtaining and confirming data from investigators.	5
Data items	11	List and define all variables for which data were sought (e.g., PICOS, funding sources) and any assumptions and simplifications made.	5
Risk of bias in individual studies	12	Describe methods used for assessing risk of bias of individual studies (including specification of whether this was done at the study or outcome level), and how this information is to be used in any data synthesis.	-
Summary measures	13	State the principal summary measures (e.g., risk ratio, difference in means).	-
Synthesis of results	14	Describe the methods of handling data and combining results of studies, if done, including measures of consistency (e.g., I^2) for each meta-analysis.	-

For peer review only

PRISMA 2009 Checklist

Section/topic	#	Checklist item	Reported on page #
Risk of bias across studies	15	Specify any assessment of risk of bias that may affect the cumulative evidence (e.g., publication bias, selective reporting within studies).	-
Additional analyses	16	Describe methods of additional analyses (e.g., sensitivity or subgroup analyses, meta-regression), if done, indicating which were pre-specified.	-
RESULTS			
Study selection	17	Give numbers of studies screened, assessed for eligibility, and included in the review, with reasons for exclusions at each stage, ideally with a flow diagram.	5
Study characteristics	18	For each study, present characteristics for which data were extracted (e.g., study size, PICOS, follow-up period) and provide the citations.	Table 1
Risk of bias within studies	19	Present data on risk of bias of each study and, if available, any outcome level assessment (see item 12).	-
Results of individual studies	20	For all outcomes considered (benefits or harms), present, for each study: (a) simple summary data for each intervention group (b) effect estimates and confidence intervals, ideally with a forest plot.	-
Synthesis of results	21	Present results of each meta-analysis done, including confidence intervals and measures of consistency.	-
Risk of bias across studies	22	Present results of any assessment of risk of bias across studies (see Item 15).	-
Additional analysis	23	Give results of additional analyses, if done (e.g., sensitivity or subgroup analyses, meta-regression [see Item 16]).	-
DISCUSSION			
Summary of evidence	24	Summarize the main findings including the strength of evidence for each main outcome; consider their relevance to key groups (e.g., healthcare providers, users, and policy makers).	11-12
Limitations	25	Discuss limitations at study and outcome level (e.g., risk of bias), and at review-level (e.g., incomplete retrieval of identified research, reporting bias).	12
Conclusions	26	Provide a general interpretation of the results in the context of other evidence, and implications for future research.	11-13
FUNDING			
Funding	27	Describe sources of funding for the systematic review and other support (e.g., supply of data); role of funders for the systematic review.	14

From: Moher D, Liberati A, Tetzlaff J, Altman DG, The PRISMA Group (2009). Preferred Reporting Items for Systematic Reviews and Meta-Analyses: The PRISMA Statement. PLoS Med 6(7): e1000097. doi:10.1371/journal.pmed1000097

For more information, visit: www.prisma-statement.org.

For peer review only

BMJ Open

Methodological Considerations for Linking Household and Healthcare Provider Data for Estimating Effective Coverage: A Systematic Review

Journal:	*BMJ Open*
Manuscript ID	bmjopen-2020-045704.R1
Article Type:	Original research
Date Submitted by the Author:	12-Apr-2021
Complete List of Authors:	Carter, Emily; Johns Hopkins University Bloomberg School of Public Health, International Health<br>Leslie, Hannah; Harvard TH Chan School of Public Health, Global Health and Population<br>Marchant, Tanya; London Sch Hyg , Disease Control<br>Amouzou, Agbessi; Johns Hopkins University Bloomberg School of Public Health, International Health<br>Munos, Melinda; Johns Hopkins University, Bloomberg School of Public Health, Department of International Health
**Primary Subject Heading**:	Global health
Secondary Subject Heading:	Research methods, Public health, Health services research, Evidence based practice
Keywords:	Public health < INFECTIOUS DISEASES, Quality in health care < HEALTH SERVICES ADMINISTRATION & MANAGEMENT, PUBLIC HEALTH, STATISTICS & RESEARCH METHODS

SCHOLARONE™
Manuscripts

I, the Submitting Author has the right to grant and does grant on behalf of all authors of the Work (as defined in the below author licence), an exclusive licence and/or a non-exclusive licence for contributions from authors who are: i) UK Crown employees; ii) where BMJ has agreed a CC-BY licence shall apply, and/or iii) in accordance with the terms applicable for US Federal Government officers or employees acting as part of their official duties; on a worldwide, perpetual, irrevocable, royalty-free basis to BMJ Publishing Group Ltd ("BMJ") its licensees and where the relevant Journal is co-owned by BMJ to the co-owners of the Journal, to publish the Work in this journal and any other BMJ products and to exploit all rights, as set out in our licence.

The Submitting Author accepts and understands that any supply made under these terms is made by BMJ to the Submitting Author unless you are acting as an employee on behalf of your employer or a postgraduate student of an affiliated institution which is paying any applicable article publishing charge ("APC") for Open Access articles. Where the Submitting Author wishes to make the Work available on an Open Access basis (and intends to pay the relevant APC), the terms of reuse of such Open Access shall be governed by a Creative Commons licence – details of these licences and which Creative Commons licence will apply to this Work are set out in our licence referred to above.

Other than as permitted in any relevant BMJ Author's Self Archiving Policies, I confirm this Work has not been accepted for publication elsewhere, is not being considered for publication elsewhere and does not duplicate material already published. I confirm all authors consent to publication of this Work and authorise the granting of this licence.

For peer review only

Methodological Considerations for Linking Household and Healthcare Provider Data for Estimating Effective Coverage: A Systematic Review

Emily D. Carter*[1], Hannah H. Leslie[2], Tanya Marchant[3], Agbessi Amouzou[1], Melinda Munos[1]

[1] Institute for International Programs, Johns Hopkins Bloomberg School of Public Health, Baltimore, MD, USA

[2] Department of Global Health and Population, Harvard T.H. Chan School of Public Health, Boston, MA, USA

[3] Faculty of Infectious and Tropical Diseases, London School of Hygiene & Tropical Medicine, London, UK

*Corresponding author: Emily Carter, Johns Hopkins Bloomberg School of Public Health, 615 N Wolfe St, Baltimore, MD, 21205, ecarter@jhu.edu

Word Count: 4998

For peer review only - http://bmjopen.bmj.com/site/about/guidelines.xhtml

ABSTRACT

Objective: To assess existing knowledge related to methodological considerations for linking population-based surveys and health facility data to generate effective coverage estimates. Effective coverage estimates the proportion of individuals in need of an intervention who receive it with sufficient quality to achieve health benefit.

Design: Systematic review of available literature.

Data sources: Medline, Carolina Population Health Center, and Demographic and Health Survey (DHS) publications and hand-search of related or referenced works of all articles included in full text review. The search included publications from 1 January 2000 to 29 March 2021.

Eligibility criteria: Publications explicitly evaluating 1) the suitability of data, 2) the implications of the design of existing data sources, and 3) the impact of choice of method for combining datasets to obtain linked coverage estimates.

Results: Of 3805 papers reviewed, 70 publications addressed relevant issues.. Limited data suggest household surveys can be used to identify sources of care, but their validity in estimating intervention need was variable. Methods for collecting provider data and constructing quality indices were diverse and presented limitations. There was little empirical data supporting an association between structural, process, and outcome quality. Few studies addressed the influence of the design of common data sources on linking analyses, including imprecise household GIS data, provider sampling design, and estimate stability. The most consistent evidence suggested under certain conditions, combining data based on geographical proximity (ecological linking) produced similar estimates to linking based on the specific provider utilized (exact-match linking).

Conclusions: Linking household and healthcare provider can leverage existing data sources to generate more informative estimates of intervention coverage and care. However, existing evidence on methods for linking data for effective coverage estimation are variable and numerous methodological questions remain. There is need for additional research to develop evidence-based, standardized best practices for these analyses.

Strengths and limitations of this study

- We systematically reviewed a wide range of methodological issues pertaining to linking population-based and health provider data for effective coverage estimation
- The review was limited by the diversity of terminology and fields related to the linking methodology
- Multiple search strategies were used to minimize the likelihood of overlooking relevant publications
- Results of the review are summarized and related to actionable items and needs for future research

For peer review only - http://bmjopen.bmj.com/site/about/guidelines.xhtml

For peer review only

For peer review only - http://bmjopen.bmj.com/site/about/guidelines.xhtml

BACKGROUND

There is growing demand for tracking progress towards the sustainable development goals (SDGs) through effective coverage estimates [1,2]. Effective coverage measures assess not only the proportion of individuals in need of an intervention who receive it, but also the content and quality of services received with an aim to estimate the proportion of individuals receiving the health benefit of an intervention [2]. Numerous publications have estimated effective coverage [3] using a range of methods and measures to define intervention need, receipt, and quality.

Linking household and health provider data is a promising means of generating effective coverage estimates that provide population-based estimates and incorporate data on service quality from health facilities. Data from household surveys can provide a population-based estimate of intervention need and care-seeking for services, such as the proportion of women with a recent live birth who delivered in a health facility. However, a number of maternal, newborn, and child health interventions [4,5] cannot be accurately measured through household surveys due to reporting errors and biases by respondents (e.g., the proportion of women who received a uterotonic during delivery). Health provider assessments yield information on provider quality, including available infrastructure, commodities, equipment, human resources, and potentially provision of care. Provider data do not capture need for care in the population, care-seeking behavior, or the experience of individuals who do not access the formal health system. Linking these two data sources can provide a more complete picture of population access to and coverage of high-quality health services, for example the proportion of women who delivered at a health facility with sufficient structural resources and competence to provide appropriate labor and delivery care.

There are many approaches for combining household and provider datasets [6]. The results depend on the choice of data and of methods for combining datasets. However, very limited guidance exists to guide decision making. We conducted a systematic review to understand the current evidence base for effective coverage linking methods and identify needs for further research.

METHODS

We searched for papers addressing methods or assumptions regarding: 1) the suitability of household and provider[1] data used in linking analyses, 2) the implications of the design of existing household (Demographic and Health Survey [DHS] and Multiple Indicator Cluster Survey [MICS]) and provider (Service Provision Assessment [SPA] and Service Availability and Readiness Assessment [SARA]) data sources commonly used in linking analyses, and 3) the impact of choice of method for combining datasets to obtain linked coverage estimates.

Our primary search was conducted in Medline. The search was limited to papers published between January 1, 2000 and March 29, 2021, that included terms related to 1) effective coverage, benchmarking, system dynamics, or universal health coverage metrics, or 2) structural, process, and/or health outcome quality, 3) linking analyses using terms adapted from Do and colleagues [6], 4) validity of self-report health indicators, and 5) spatial methods for measuring utilization or distance to care. A full list of Medline search terms and PRISMA checklist are presented in Supplementary File 1 and 2,

[1] We define health providers as health care outlets such as health facilities, pharmacies, and community-based health workers

For peer review only - http://bmjopen.bmj.com/site/about/guidelines.xhtml

respectively. The search was conducted using English-language terms; however, publications in English, Spanish, and French were reviewed if captured in the search. Additionally, we conducted searches using these criteria in Population Health Metrics (which was not fully indexed in Medline at the time of our search), the Carolina Population Health Center, and DHS publications. In a second step, we hand-searched the references of a systematic review by Do and colleagues on linking household and facility data to estimate coverage of reproductive, maternal, newborn, and child health services [6], and a review by Amouzou and colleagues of effective coverage analyses [3]. Both the Do and Amouzou reviews summarized publications that linked data or estimated effective coverage; however, they did not systematically address methodological concerns or relevant results for guiding application of these methods. We also hand-searched the references, citing works, and journal- or database interface-generated related publications of all articles that passed the title and abstract review. Particularly relevant papers published after the formal search date were included in the review if captured through these snowball review mechanisms.

Publications were reviewed for relevant analyses or commentary related to linking methodologies. Articles were included if they explicitly evaluated or compared assumptions used in linking approaches for at least one of the areas defined above. The review focused on low- and middle-income countries (LMICs) and data sources common in these settings, however publications from high-income settings were retained if the relevant evidence could translate to LMICs (e.g., use of centroid GPS location in estimates of distance, validity of provider quality measures). No formal quality assessment was conducted due to the diversity of study designs and research objectives of the papers relevant to the review. Title and abstract review were conducted simultaneously by the first author (EC). Data extraction included the title, author, year of publication, country or countries included in analysis, data source, and specific analyses or findings relevant to linking loosely categorized by topic areas. Topical area groupings emerged from the review and were used to structure the findings.

Patient and public involvement

As a systematic review, neither patients nor the public were involved in the design, conduct, reporting, or dissemination plans of our research.

RESULTS

The Medline search produced 3669 publications, along with 79 from the Carolina Population Center, 4 from Population Health Metrics, 12 DHS publications, 35 papers included in the review by Amouzou and colleagues and 49 papers included in the review by Do and colleagues meeting the publication date restrictions. After removing duplicates, 3805 publications were included in the title and abstract review and 236 were included in the full text review. Of those papers included in the full text review, 56 publications addressed a methodological concern related to linking household and provider data and were included in the final review. Fourteen additional publications were identified through the snowball review of references and related works (Fig 1 – PRISMA flow diagram). In total 70 publications addressed one or more methodological concern, including the suitability of household (n=13) and provider data (n=39) for use in linking analyses, concerns related to the design of existing household (n=6) and provider (n=4) data sources, and methods for combining household and facility data (n=14). A list of publications included in the review and a summary of their contributions to the review are provided in Table 1.

For peer review only - http://bmjopen.bmj.com/site/about/guidelines.xhtml

-TABLE 1-

Suitability of household and provider data for linking analyses

Suitability of household data needed for linked estimates

In effective coverage linking analyses, household surveys can be used to estimate the proportion of the population in need of healthcare, as well as care-seeking behavior. Household surveys must produce valid estimates of these parameters and provide care-seeking data that can be linked to provider assessments. This review identified papers discussing issues in defining intervention need (n=8) and care-seeking (n=5) that should guide selection of indicators for linking.

Intervention need

Estimation of intervention need may require solely population demographics such as age (e.g., for prevention and health promotion interventions) or may require defining specific illnesses or conditions. The latter is more subject to reporting bias [7]. Multiple studies have shown poor association or biases between maternally-reported symptoms and clinical pneumonia [8,9], malaria [10], and diarrhea [11] in children under five. A handful of studies (n=3) showed maternal report of both maternal and newborn birth complications is variable [12–14]. A simulation by Shengelia and colleagues demonstrated the effect of the divergence of true from perceived intervention need on effective coverage estimates. The authors propose estimating the posterior probability of disease based on responses to symptomatic questions using a Bayesian model to measure disease presence on a probabilistic scale [7]. However, there has been no work on how to integrate these adjusted estimates into effective coverage estimates.

Care-seeking behavior

Four studies addressed the accuracy of respondent report on seeking care. Mothers in Zambia and Mozambique were able to accurately report on the type of health provider where they sought sick child [15] and delivery care [16], respectively. However, studies in two countries suggested women cannot report on the type of health worker who attended to them during labor and delivery and immediate post-natal care [17,18]. Wang and colleagues note that provider categories are not standardized between population surveys and health system assessments, with population surveys often including vague or overly broad categories that do not directly match SPA/SARA categories and require harmonization [19].

Suitability of healthcare quality data needed for linked estimates

Provider assessments present data on service content and quality for effective coverage linking analyses. However, the measurement, construction, and interpretation of provider quality measures are highly variable and may significantly alter effective coverage estimates. This paper does not present an exhaustive review of healthcare quality measures or the association between levels of quality. A comprehensive summary of quality of care concepts and measurement approaches, along with their relative strengths and limitations, was presented by Hanefeld and colleagues [20]. Publications of particular relevance to linking analyses are noted here, with an emphasis on national provider survey data as the most common source of provider data for linking analyses.

Methods used in assessing provider quality

For peer review only - http://bmjopen.bmj.com/site/about/guidelines.xhtml

A review by Nickerson and colleagues found significant variability in the data collected and methods used in health facility assessment tools in LMICs [21]. While SPA and SARA data are the most widely used sources of data on health service delivery in LMICs, one paper noted that these surveys focused primarily on structural quality with less data on provision and experience of care [22]. The lack of process quality data is in part related to the reliance on direct observation of clinical care – a time- and resource-intensive method – to collect these data. None of the studies included in the review used HMIS data to generate linked coverage estimates. A desk review by MCSP found that data collected through HMIS was variable across countries, data recorded within registers often was not transmitted through the system, and only a limited number of indicators collected were related to the provision of health services [23].

Nine publications assessed alternatives to direct observation of clinical care for collecting process quality data. Two studies found no association between process quality and maternal perceptions of the quality of care received [24,25] while one study found perceived quality was associated with the number of services received but not structural quality [26]. Agreement between observed care and health records or provider report was also variable [27–29]. A review by Hrisos and colleagues found few studies to support use of patient report, provider self-report, or record review as proxy measures of clinical care quality [30]. In the US, vignettes performed better than chart abstraction for estimating quality [31]. Another review found providers were unable to accurately assess their own performance, with the worst accuracy among the least skilled providers [32]. Five other publications used alternative methods for measuring process quality, including use of vignettes [26,33], register review [26,34], most recent delivery interview [35], and an mHealth tool [36], but did not assess their performance against other measurement methods.

Content of provider quality indices

Most linking papers estimating effective coverage included in this review (n=15) characterized provider quality using structural measures of quality, with or without measures of process quality. Various approaches were used to select items for inclusion in these measures. Measures of structural and process quality were derived from either national or international guidance on minimum service availability and required commodities, equipment, infrastructure, training, or actions. Measures used by effective coverage analyses included SPA or SARA structural indicators [33,37–39] and/or clinical observations [38,40–43], emergency obstetric and newborn care (EmONC) functions [28,34,44,45], provider recall of actions during their last delivery [35,44], and measured health outcomes [46,47].

Construction of provider quality indices

In addition to the range of variables used in provider measures, there was no consensus on the approach to use to select and combine variables to generate quality indices. The reviewed publications used a variety of approaches to construct indices including weighted indices [41], simple averages across all indicators or domains [33,39,40,42–44], and categorization using set thresholds or relative categories [26,37,45]. A review of quality measurement using SPA data found that studies frequently did not apply a theoretical framework when selecting indicators for quality measures, and that there was high variability in the indicators included in quality scores [48]. In our review, seven publications presented data on the performance of different measurement modes and summary approaches. Two studies found the method of selecting and combining quality indicators had little effect on overall effective coverage estimates [49,50]. However, two other studies found inconsistency in the rankings of health

For peer review only - http://bmjopen.bmj.com/site/about/guidelines.xhtml

facilities when using different index methods [51,52]. Two studies using PCA to create SPA health service indices found the reduced indices explained only a limited amount of the variance across indicators [52,53]. An analysis of SPA data in ten countries found indices empirically-derived through machine learning captured a large proportion of the service readiness data in the full SPA index, however the selected set of indicators varied across countries, and an index generated through expert review captured very little of the data from the full index [54]. Two studies found that few facilities could meet all requirements when applying a threshold, limiting the utility of the approach [45,51].

Performance of provider measures

Despite the common usage of SPA and SARA data-derived structural and process quality measures, the review found limited data explicitly assessing the association of these measures with each other and health outcomes (n=7). Three studies, two incorporating data from multiple countries, found little association between structural quality and process quality [41,55,56]. However, an analysis of SPA data from three countries found a small but significant association between antenatal care (ANC) facility structural and process quality and suggests structural quality can limit provider performance when basic infrastructure and commodities are unavailable [50]. Akachi and Kruk emphasized the limited number of studies showing process quality associated with health outcomes [57]. Two studies in Malawi found a small association between an obstetric quality index and decreased neonatal mortality [58] and an association between quality-adjusted ANC nutrition intervention coverage and decreased low birthweight prevalence [42]. Another found a national UHC "heath service coverage" index correlated strongly with infant mortality rate and life expectancy [59].

Implications of design of existing household and provider data sources commonly used in linking analyses

Issues related to household and cluster location data

The way in which common household surveys, particularly the DHS and MICS, collect and process location data may also impact the validity of some linked estimates. In many household datasets used for linked analyses, the precise location of individual households is often unknown. The DHS collects central point locations for clusters, rather than household locations, and displaces these points in publicly released datasets [60]. MICS often does not collect or make GIS data available [61]. Imprecision around household location may influence the accuracy of estimates generated by linking household and provider data based on geographic proximity.

Data on household location

The effect of using cluster central point locations (centroids) rather than individual household locations in linking analyses was not addressed by any publication identified in this review. However, four studies looked at the effect of using centroids of varying areal units versus household locations in distance analyses. Two studies found using US census tract [62] and zip-code [63] centroid locations produced little difference in measures of facility access compared to household location. A third study showed use of areal unit centroids resulted in misclassification of household access to health-related facilities, especially in less densely populated rural areas [64]. However, in rural Ghana, measures calculated from village centroids identified the same closest facility as measures from compound locations for over 85% of births [65].

For peer review only - http://bmjopen.bmj.com/site/about/guidelines.xhtml

Cluster displacement

Displacement of cluster central points might induce additional error in analyses based on geographic proximity. A DHS analytic report found that ignoring DHS displacement in analyses that used distance to a resource as a covariate resulted in increased bias and mean squared error (MSE). However, this will not affect linking by administrative unit because DHS has restricted displacement to within the representative sample administrative unit since 2009 [60]. A simulation analysis in Rwanda reported DHS cluster displacement produced less misclassification in level of access and relative service quality than healthcare provider sampling [66].

Issues related to provider sampling

Typical sampling designs for healthcare provider data also present issues for linking analyses. Both SPAs and SARAs are sampled independently of household surveys, thus, there may be no sampled facilities near household survey clusters [67]. SPA and SARA surveys typically collect data on a sample, rather than census, of public, private, and NGO health facilities and exclude non-facility providers, such as pharmacies or community health workers. In most settings, facilities are sampled and analyzed to be representative of all facilities within a managing authority, level, and/or geographic area, and the results of the provider assessment are not intended to represent the population utilizing health services [67]. For provider assessments conducting direct observations of clinical care, the number and type of interactions observed within each health facility is dependent on patient volume and chance.

Provider sampling frame

Two papers assessed the impact of excluding non-facility providers on linked effective coverage estimates. In Zambia and Cote d'Ivoire, CHWs offered a level of care for sick children similar to first-level public facilities. Excluding these providers reduced estimates of effective coverage in Zambia where CHWs were a significant source of skilled care in rural areas estimates [33], but had little effect in Cote d'Ivoire where they were an insignificant source of care [40]. In both studies, exclusion of pharmacies did not alter effective coverage estimates as they were an uncommon source of care, though they offered moderate structural quality [33,40].

Provider sampling design

Two publications addressed the impact of facility survey sampling designs. At the facility level, Skiles and colleagues' analysis demonstrated that sampling facilities, rather than using a census, led to an underestimation of the adequacy of the health service environment and substantial misclassification error in relative service environment for individual clusters [66]. No studies addressed the suitability of SPA or SARA facility sampling strategies for generating stable quality estimates for use in linking analyses at a level below administrative unit used for the sampling approach.

A Measure Evaluation manual emphasized that data on provision of services (collected through observation of client-staff interactions), experience of care (collected through client exit interviews), and staff characteristics (collected through health worker interviews) are sampled independently and collected among health workers and care interactions available on the day of the survey. These data are a sub-sample of the overall survey and representative at the level the survey is sampled to be representative – not at the facility level [67]. This paper proposed multiple linked sampling approaches to capture geographically concordant household and provider data for linked analyses. While multiple

For peer review only - http://bmjopen.bmj.com/site/about/guidelines.xhtml

studies included in this review used a census or sample of providers derived from a household sample, none implemented this approach at a national scale.

Issues related to timing of surveys used in linked coverage estimates

Both care-seeking behavior and provider quality are likely to vary over time, and both household and provider surveys are conducted infrequently in LMICs (~3-5 years). Linked coverage estimates for RMNCH may cover a long timeframe as the reference period for care-seeking in household surveys varies from 2 weeks (sick child care) to 2-5 years (peripartum care). Population movement and quality improvement efforts at facilities further complicate associations with increasing time lags. The implications of linking household and provider indicators of different temporal periods is unclear.

Stability of provider indicators

No paper in this review specifically addressed the effect of provider indicator stability on linked effective coverage estimates. However, three linking papers presented data on the stability of some health facility indicators over time. EQUIP studies in Uganda and Tanzania found moderate variability in the availability of some maternal and newborn health commodities and services over a 2-3 year period [44,68,69].

Stability of household indicators

Care-seeking behavior, including overall rates of care and utilization of different sources of care, may also change over time. Analysis of care-seeking for child illness [70] and maternal healthcare [71] in multiple low- and middle-income countries over time showed high inconsistency in trends across countries. However, no identified studies addressed the consequences of this temporal variability within the context of linking analyses.

Impact of choice of method for combining household and provider data

The approach for combining household and provider data can potentially have a significant impact on linked coverage estimates. Methods used to link data, including exact-match and various types of ecological linking, are defined in Table 2. Exact-match linking assigns provider information to individuals in the target population based on their specific source of care. This approach, while potentially subject to the reporting biases described previously, is considered the most precise approach for combining the two data sets in the absence of individual patient health records [6]. Without data on specific source of care, ecological linking approaches are designed to approximate care-seeking behavior or model healthcare access by linking the target population to sources of care based on geographic proximity or administrative catchment area, making assumptions about service access and use.

-TABLE 2-

Comparison of exact-match and ecological linking methods for estimating effective coverage

Three publications explicitly compared effective coverage estimates generated using exact-match and ecological linking methods (Table 3) [33,40,44]. Estimates generated using the exact-match linking approach were considered the gold-standard measure of effective coverage. All three publications found exact-match linked effective coverage estimates were similar to straight-line [33,40], travel time [33,40], 5-km buffer [33], 10-km buffer [40], and administrative unit [33,40,44] geolinked estimates for antenatal [40], labor and delivery [40,44], postnatal [40] and sick child [33,40] care when linking was

For peer review only - http://bmjopen.bmj.com/site/about/guidelines.xhtml

restricted by the reported provider category (e.g., hospital, health center, community health worker). Distance-restricted linking approaches, such as linking to providers within a 5 km radius, produced inaccurate results if unlinked events were treated as no care [33]. Restriction of geographic linking to only providers within the reported category of care and/or weighting by providers' relative patient volume improved agreement between the exact-match and ecological linking estimates [40,44]. All three studies also used provider data obtained from a census of health facilities, and therefore the findings may not be applicable when household data are linked to a sample survey of health facilities.

-TABLE 3-

Performance of measures of geographic proximity for ecological linking

Eight studies assessed the performance of geographic measures in assigning households or individuals to their reported source of healthcare. Four studies in sub-Saharan Africa compared the predicted source of care based on geographic proximity against the true source of care. They found straight-line and road distance performed similarly [72], high performance of shortest travel time method [73], and better performance of straight-line distance compared to road distance [33,40]. In the US, a more sophisticated approach (2-stage and 3-stage floating catchment area) performed better than alternatives methods in assigning households to their source of care [74]. Three studies in sub-Saharan Africa evaluated use of Theissen boundaries, a method of defining catchment boundaries based on the optimal distance between known providers, in assigning households to the catchment of facilities they utilized. The studies found high performance in some settings [75], but poorer performance related to the use of higher-order facilities [76] and influence of public transportation routes [77].

Statistical challenges

Most linking analyses that have generated effective coverage estimates by assigning individuals the quality score of the reported or linked source of care have derived estimates of uncertainty based on household sampling error and ignored any sampling error around provider data. However, two analyses used the Delta method [78] for estimating the variance of effective coverage estimates generated by multiplying service use and readiness [19,44]. A simulation study compared three variance estimation methods for linked effective coverage measures (household sampling error alone, parametric bootstrapping, and the delta method), and found that all three performed similarly for large samples. However, the delta method produced more valid confidence bounds with smaller samples or when the effective coverage estimate approached either 0 or 100% [79].

DISCUSSION

This review found a variable number of publications that addressed the diverse methodological issues related to linking household and provider datasets. A summary of key findings and needs for further research is presented in table 4 and discussed below.

-TABLE 4-

Suitability of household and provider data for linking analyses

For peer review only - http://bmjopen.bmj.com/site/about/guidelines.xhtml

We identified a number of papers that critically assessed household and provider data needed for linking analyses. The limited existing data on respondent-reported care-seeking suggest respondents can identify sources of care if not individual healthcare worker cadre, but additional validation in various settings and service areas, such as postnatal care, would be informative. Further, it is essential to ensure that categorization of sources of care in household surveys align with the categories used in provider assessments to facilitate linking datasets. The validity of household survey data for estimating populations in need was more variable. While some populations in need can be clearly defined, others, particularly those requiring symptom-derived diagnoses based on respondent report, have demonstrated potential for bias. Additional work is needed to explore alternative methods for identifying populations in need in population-based data collection.

The content and construction of provider quality indices was highly variable across publications, but largely derived from facility surveys and was informed by international guidelines or recommendations. Methods for collecting provider quality have a number of limitations, and no single method perfectly encompasses all aspects of care [80]. The review found a lack of agreement between measures of quality derived through various means of collection. Overall, there was little empirical data supporting association between structural quality and process quality, and measures of quality and appropriate care or good health outcomes, although the number of reviewed studies was very limited. However, as articulated by Nguhiu and colleagues, there is need to consider quality indicators' "intrinsic value as levers for management action" and application to policy decision-making in addition to their ability to capture or predict associated health gain [38]. Many important indicators of healthcare quality, particularly around patient-centered care, are not currently measured through existing tools and there is a need to better capture these indicators [81,82]. Additional research is needed in the short term to develop and evaluate new quality indices utilizing existing data sources (e.g., facility surveys, HMIS, and medical records) with an aim of identifying a standardized approach for selecting, combining, and interpreting indicators that reflect aspects of provider quality necessary for delivering appropriate, respectful, and effective care. Longer term, substantial effort is needed to strengthen or adapt existing mechanisms or develop alternative methods for collecting provider quality indicators that can produce timely and informative estimates for tracking effective coverage of key interventions.

Implications of the design of existing household and provider data sources commonly used in linking analyses

Few studies addressed the influence of the design of common data sources on linking analyses, including the impact of imprecise household GIS data, provider sampling frame and sampling design, and estimate stability. However, there was a lack of concrete evidence around the impact of these factors on linked effective coverage estimates. Explicitly evaluating the impact of imprecise household location, sampling design, and temporal gaps between measures within the context of effective coverage estimation would be informative. Mixed results on the inclusion of non-facility providers in provider assessments for effective coverage estimation emphasize the need to empirically assess the utilization and service quality of non-facility providers in a given setting prior to conducting a linking analysis, as the quality and use of these providers varies by health area and setting [70,71,83]. Although data related to impact on effective coverage estimation were limited, small samples of client-staff observations, sampling of healthworkers and facilities, and temporal gaps between household and provider data have the potential to bias estimates. The available data suggest that developing and testing alternative means of sampling health providers could improve the validity of linked estimates of effective coverage, including

For peer review only - http://bmjopen.bmj.com/site/about/guidelines.xhtml

evaluating joint sampling approaches proposed by Measure Evaluation [67] or used by other data collection mechanisms such as PMA2020 and the India District Level Household and Facility Survey.

Impact of choice of method for combining household and provider data

The most consistent evidence found through the review was around methods for combining data sets. Three papers compared ecological and exact match linking and reported that ecological linking (when accounting for frequency of provider utilization by type) produced similar estimates to exact-match linking. The agreement between the three publications that compared exact-match and ecological linking is promising. Exact-match linking is considered the most precise method for generating linked estimates; however, ecological linking is often more feasible because it does not require information on exact source of care or data on all providers. The papers further point to the need to maintain data on type of provider from which care was sought or the relative volume of patients seen by providers in order to generate valid estimates of effective coverage. All three studies were conducted in rural sub-Saharan Africa in settings with high utilization of public sector health facilities; additional studies evaluating the performance of these methods in settings with a more diverse healthcare landscape would be informative. Other papers evaluated ecological linking approaches and found similar estimates of access to care or effective coverage using different approaches for assessing geographic proximity, although the ability of methods to capture true source of care was more variable. External to this review, additional data suggests individuals may not always utilize the closest source of care and may bypass providers in favor of providers offering better care [37,84,85]. These findings along with the analyses comparing exact-match and ecological linking approaches emphasize the need to carefully select methods for performing ecological linking and to control for true care-seeking behavior as much as possible by accounting for the type of provider from which care was sought or weighting by utilization in linking analyses. There is also need to further develop approaches and tools for estimating uncertainty around linked effective coverage estimates.

Evidence across the review demonstrates the need for careful choice of methods, data sources, and indicators when conducting studies or analyses to link household and provider data for effective coverage estimation. An exploration of the precise effect of setting characteristics, such as variation in provider quality, on effective coverage estimates is needed to guide decision-making in the selection of linking methods. Once more of these issues have been evaluated, additional tools and guidance to facilitate use of these methods will be needed.

The review was limited by the diversity of terminology and fields related to the linking methodology. However, the use of multiple search strategies minimized likelihood of overlooking relevant publications. No formal grading of publication quality was included in the assessment, but the choice to conduct the search through Medline was intended to ensure a basic level of quality across the diverse study designs included in the review. Additionally, the diversity of fields, approaches, and questions made it difficult to summarize the findings neatly, emphasizing the need for communication between researchers, more standard terminology, and, ideally, a cohesive research strategy going forward. Recent efforts have aimed to align definitions of effective coverage [2]. We attempt in table 4 to translate the review results into actionable items and needs for future research.

CONCLUSIONS

For peer review only - http://bmjopen.bmj.com/site/about/guidelines.xhtml

Linking household and healthcare provider data is a promising approach that leverages existing data sources to generate more informative estimates of intervention coverage and care. These methods can potentially address limitations of both household and provider surveys to generate population-based estimates that reflect not only use of services, but also the content and quality of care received and the potential for health benefit. However, there is need for additional research to develop evidence-based, standardized best practices for these analyses. The most pressing priorities identified in this review are: 1) for those collecting data from health systems to explore methods to strengthen existing provider data collection mechanisms and promote temporal and geographic alignment with population-based measures, 2) for those collecting population-based data to address validity of self-reported intervention need and ensure indicators of access and utilization of care are measured to facilitate linking analyses, and 3) for those conducting linked analyses to standardize approaches for generating and interpreting effective coverage indicators, including sources of uncertainty, to ensure we are producing evidence that is harmonized, informative, and actionable for governments and valid for monitoring population health globally.

For peer review only

Author contributions: Conceived of the study design: EC MM. ICMJE criteria for authorship read and met: EC HL TM AA MM. Agree with manuscript and conclusions: EC HL TM AA MM. Conducted review: EC. Drafted paper: EC. All authors read, edited, and approved the manuscript.

Competing Interests: We declare no competing interests in accordance with ICMJE Conflict of Interest Guidelines.

Funding: This work was supported by the Improving Coverage Measurement grant (OPP1172551) from the Bill & Melinda Gates Foundation. The funders did not have any role in the design of the study and collection, analysis, and interpretation of data or in writing the manuscript.

Data Sharing: As a review article, this article reports data from previously published studies.

Ethics Approval: Ethical approval was not required as this review only included publicly available, published data.

Patient and public involvement Patients and/or the public were not involved in the design, or conduct, or reporting, or dissemination plans of this research.

Patient consent for publication Not required.

Provenance and peer review Not commissioned; externally peer reviewed.

For peer review only

For peer review only - http://bmjopen.bmj.com/site/about/guidelines.xhtml

REFERENCES

1 Ng M, Fullman N, Dieleman JL, *et al.* Effective coverage: a metric for monitoring Universal Health Coverage. *PLoS Med* 2014;**11**:e1001730. doi:10.1371/journal.pmed.1001730

2 Marsh AD, Muzigaba M, Diaz T, *et al.* Effective coverage measurement in maternal, newborn, child, and adolescent health and nutrition: progress, future prospects, and implications for quality health systems. *Lancet Glob Health* 2020;**8**:e730–6. doi:10.1016/S2214-109X(20)30104-2

3 Amouzou A, Leslie HH, Ram M, *et al.* Advances in the measurement of coverage for RMNCH and nutrition: from contact to effective coverage. *BMJ Global Health* 2019;**e001297**.

4 Bryce J, Arnold F, Blanc A, *et al.* Measuring Coverage in MNCH: New Findings, New Strategies, and Recommendations for Action. *PLOS Med* 2013;**10**:e1001423. doi:10.1371/journal.pmed.1001423

5 Day LT, Sadeq-Ur Rahman Q, Ehsanur Rahman A, *et al.* Assessment of the validity of the measurement of newborn and maternal health-care coverage in hospitals (EN-BIRTH): an observational study. *Lancet Glob Health* 2021;**9**:e267–79. doi:10.1016/S2214-109X(20)30504-0

6 Do M, Micah A, Brondi L, *et al.* Linking household and facility data for better coverage measures in reproductive, maternal, newborn, and child health care: systematic review. *J Glob Health* 2016;**6**:020501. doi:10.7189/jogh.06.020501

7 Shengelia B, Tandon A, Adams OB, *et al.* Access, utilization, quality, and effective coverage: an integrated conceptual framework and measurement strategy. *Soc Sci Med 1982* 2005;**61**:97–109. doi:10.1016/j.socscimed.2004.11.055

8 Ayede AI, Kirolos A, Fowobaje KR, *et al.* A prospective validation study in South-West Nigeria on caregiver report of childhood pneumonia and antibiotic treatment using Demographic and Health Survey (DHS) and Multiple Indicator Cluster Survey (MICS) questions. *J Glob Health* 2018;**8**:020806. doi:10.7189/jogh.08-020806

9 Hazir T, Begum K, El Arifeen S, *et al.* Measuring coverage in MNCH: a prospective validation study in Pakistan and Bangladesh on measuring correct treatment of childhood pneumonia. *PLoS Med* 2013;**10**:e1001422. doi:10.1371/journal.pmed.1001422

10 D'Acremont V, Lengeler C, Genton B. Reduction in the proportion of fevers associated with Plasmodium falciparum parasitaemia in Africa: a systematic review. *Malar J* 2010;**9**:240. doi:10.1186/1475-2875-9-240

11 Walker CLF, Fontaine O, Black RE. Measuring Coverage in MNCH: Current Indicators for Measuring Coverage of Diarrhea Treatment Interventions and Opportunities for Improvement. *PLOS Med* 2013;**10**:e1001385. doi:10.1371/journal.pmed.1001385

12 Keenan K, Hipwell A, McAloon R, *et al.* Concordance between maternal recall of birth complications and data from obstetrical records. *Early Hum Dev* 2017;**105**:11–5. doi:10.1016/j.earlhumdev.2017.01.003

For peer review only - http://bmjopen.bmj.com/site/about/guidelines.xhtml

13 Zimmerman LA, Shiferaw S, Seme A, *et al.* Evaluating consistency of recall of maternal and newborn care complications and intervention coverage using PMA panel data in SNNPR, Ethiopia. *PLoS ONE* 2019;**14**. doi:10.1371/journal.pone.0216612

14 Chang KT, Mullany LC, Khatry SK, *et al.* Validation of maternal reports for low birthweight and preterm birth indicators in rural Nepal. *J Glob Health* 2018;**8**.

15 Carter ED, Ndhlovu M, Munos M, *et al.* Validity of maternal report of care-seeking for childhood illness. *J Glob Health* 2018;**8**:010602. doi:10.7189/jogh.08.010602

16 Stanton CK, Rawlins B, Drake M, *et al.* Measuring Coverage in MNCH: Testing the Validity of Women's Self-Report of Key Maternal and Newborn Health Interventions during the Peripartum Period in Mozambique. *PLOS ONE* 2013;**8**:e60694. doi:10.1371/journal.pone.0060694

17 Blanc AK, Diaz C, McCarthy KJ, *et al.* Measuring progress in maternal and newborn health care in Mexico: validating indicators of health system contact and quality of care. *BMC Pregnancy Childbirth* 2016;**16**:255. doi:10.1186/s12884-016-1047-0

18 Blanc AK, Warren C, McCarthy KJ, *et al.* Assessing the validity of indicators of the quality of maternal and newborn health care in Kenya. *J Glob Health* 2016;**6**:010405. doi:10.7189/jogh.06.010405

19 Wang W, Mallick L, Allen C, *et al.* Effective coverage of facility delivery in Bangladesh, Haiti, Malawi, Nepal, Senegal, and Tanzania. Published Online First: 1 August 2018.https://dhsprogram.com/publications/publication-as65-analytical-studies.cfm (accessed 17 May 2019).

20 Hanefeld J, Powell-Jackson T, Balabanova D. Understanding and measuring quality of care: dealing with complexity. *Bull World Health Organ* 2017;**95**:368.

21 Nickerson JW, Adams O, Attaran A, *et al.* Monitoring the ability to deliver care in low- and middle-income countries: a systematic review of health facility assessment tools. *Health Policy Plan* 2015;**30**:675–86. doi:10.1093/heapol/czu043

22 Sheffel A, Karp C, Creanga AA. Use of Service Provision Assessments and Service Availability and Readiness Assessments for monitoring quality of maternal and newborn health services in low-income and middle-income countries. *BMJ Glob Health* 2018;**3**. doi:10.1136/bmjgh-2018-001011

23 Maternal and Child Survival Program (MCSP). What Data on Maternal and Newborn Health Do National Health Management Information Systems Include? 2018. https://www.mcsprogram.org/resource/what-data-on-maternal-and-newborn-health-do-national-health-management-information-systems-include/ (accessed 20 May 2019).

24 Diamond-Smith N, Sudhinaraset M, Montagu D. Clinical and perceived quality of care for maternal, neonatal and antenatal care in Kenya and Namibia: the service provision assessment. *Reprod Health* 2016;**13**:92. doi:10.1186/s12978-016-0208-y

25 Getachew T, Abebe SM, Yitayal M, *et al.* Assessing the quality of care in sick child services at health facilities in Ethiopia. *BMC Health Serv Res* 2020;**20**:574. doi:10.1186/s12913-020-05444-7

For peer review only - http://bmjopen.bmj.com/site/about/guidelines.xhtml

26 Larson E, Hermosilla S, Kimweri A, *et al.* Determinants of perceived quality of obstetric care in rural Tanzania: a cross-sectional study. *BMC Health Serv Res* 2014;**14**:483. doi:10.1186/1472-6963-14-483

27 Hoogenboom G, Thwin MM, Velink K, *et al.* Quality of intrapartum care by skilled birth attendants in a refugee clinic on the Thai-Myanmar border: a survey using WHO Safe Motherhood Needs Assessment. *BMC Pregnancy Childbirth* 2015;**15**:17. doi:10.1186/s12884-015-0444-0

28 Nesbitt RC, Lohela TJ, Manu A, *et al.* Quality along the Continuum: A Health Facility Assessment of Intrapartum and Postnatal Care in Ghana. *PLOS ONE* 2013;**8**:e81089. doi:10.1371/journal.pone.0081089

29 Osen H, Chang D, Choo S, *et al.* Validation of the World Health Organization tool for situational analysis to assess emergency and essential surgical care at district hospitals in Ghana. *World J Surg* 2011;**35**:500–4. doi:10.1007/s00268-010-0918-1

30 Hrisos S, Eccles MP, Francis JJ, *et al.* Are there valid proxy measures of clinical behaviour? a systematic review. *Implement Sci* 2009;**4**:37. doi:10.1186/1748-5908-4-37

31 Peabody JW, Luck J, Glassman P, *et al.* Comparison of Vignettes, Standardized Patients, and Chart Abstraction: A Prospective Validation Study of 3 Methods for Measuring Quality. *JAMA* 2000;**283**:1715–22. doi:10.1001/jama.283.13.1715

32 Davis DA, Mazmanian PE, Fordis M, *et al.* Accuracy of Physician Self-assessment Compared With Observed Measures of Competence: A Systematic Review. *JAMA* 2006;**296**:1094–102. doi:10.1001/jama.296.9.1094

33 Carter ED, Ndhlovu M, Eisele TP, *et al.* Evaluation of methods for linking household and health care provider data to estimate effective coverage of management of child illness: results of a pilot study in Southern Province, Zambia. *J Glob Health* 2018;**8**:010607. doi:10.7189/jogh.08.010607

34 Wilunda C, Oyerinde K, Putoto G, *et al.* Availability, utilisation and quality of maternal and neonatal health care services in Karamoja region, Uganda: a health facility-based survey. *Reprod Health* 2015;**12**:30. doi:10.1186/s12978-015-0018-7

35 Marchant T, Tilley-Gyado RD, Tessema T, *et al.* Adding Content to Contacts: Measurement of High Quality Contacts for Maternal and Newborn Health in Ethiopia, North East Nigeria, and Uttar Pradesh, India. *PLOS ONE* 2015;**10**:e0126840. doi:10.1371/journal.pone.0126840

36 Mboya D, Mshana C, Kessy F, *et al.* Embedding systematic quality assessments in supportive supervision at primary healthcare level: application of an electronic Tool to Improve Quality of Healthcare in Tanzania. *BMC Health Serv Res* 2016;**16**:578. doi:10.1186/s12913-016-1809-4

37 Kanyangarara M, Munos MK, Walker N. Quality of antenatal care service provision in health facilities across sub-Saharan Africa: Evidence from nationally representative health facility assessments. *J Glob Health* 2017;**7**:021101. doi:10.7189/jogh.07.021101

38 Nguhiu PK, Barasa EW, Chuma J. Determining the effective coverage of maternal and child health services in Kenya, using demographic and health survey data sets: tracking progress towards universal health coverage. *Trop Med Int Health* 2017;**22**:442–53. doi:10.1111/tmi.12841

For peer review only - http://bmjopen.bmj.com/site/about/guidelines.xhtml

For peer review only

39 Chou VB, Walker N, Kanyangarara M. Estimating the global impact of poor quality of care on maternal and neonatal outcomes in 81 low- and middle-income countries: A modeling study. *PLoS Med* 2019;**16**:e1002990. doi:10.1371/journal.pmed.1002990

40 Munos M, Maiga A, Do M, *et al.* Linking household survey and health facility data for effective coverage measures: a comparison of ecological and individual linking methods using the Multiple Indicator Cluster Survey in Côte d'Ivoire. *J Glob Health* 2018;**8**.

41 Leslie HH, Sun Z, Kruk ME. Association between infrastructure and observed quality of care in 4 healthcare services: A cross-sectional study of 4,300 facilities in 8 countries. *PLoS Med* 2017;**14**:e1002464. doi:10.1371/journal.pmed.1002464

42 Joseph NT, Piwoz E, Lee D, *et al.* Examining coverage, content, and impact of maternal nutrition interventions: the case for quality-adjusted coverage measurement. *J Glob Health* 2020;**10**:010501. doi:10.7189/jogh.10.010501

43 Yakob B, Gage A, Nigatu TG, *et al.* Low effective coverage of family planning and antenatal care services in Ethiopia. *Int J Qual Health Care J Int Soc Qual Health Care* 2019;**31**:725–32. doi:10.1093/intqhc/mzy251

44 Willey B, Waiswa P, Kajjo D, *et al.* Linking data sources for measurement of effective coverage in maternal and newborn health: what do we learn from individual- vs ecological-linking methods? *J Glob Health* 2018;**8**:010601. doi:10.7189/jogh.08.010601

45 Gabrysch S, Simushi V, Campbell OMR. Availability and distribution of, and geographic access to emergency obstetric care in Zambia. *Int J Gynecol Obstet* 2011;**114**:174–9. doi:10.1016/j.ijgo.2011.05.007

46 Leslie HH, Doubova SV, Pérez-Cuevas R. Assessing health system performance: effective coverage at the Mexican Institute of Social Security. *Health Policy Plan* 2019;**34**:ii67–76. doi:10.1093/heapol/czz105

47 Servan-Mori E, Avila-Burgos L, Nigenda G, *et al.* A Performance Analysis of Public Expenditure on Maternal Health in Mexico. *PloS One* 2016;**11**:e0152635. doi:10.1371/journal.pone.0152635

48 Moucheraud C, McBride K. Variability in Health Care Quality Measurement among Studies Using Service Provision Assessment Data from Low- and Middle-Income Countries: A Systematic Review. *Am J Trop Med Hyg* 2020;**103**:986–92. doi:10.4269/ajtmh.19-0644

49 Lozano R, Soliz P, Gakidou E, *et al.* Benchmarking of performance of Mexican states with effective coverage. *Lancet Lond Engl* 2006;**368**:1729–41. doi:10.1016/S0140-6736(06)69566-4

50 Sheffel A. Association between Antenatal Care Facility Readiness and Provision of Care at the Individual and Facility Level in Haiti, Malawi, and Tanzania. 2018.

51 Fisseha G, Berhane Y, Worku A, *et al.* Quality of the delivery services in health facilities in Northern Ethiopia. *BMC Health Serv Res* 2017;**17**:187. doi:10.1186/s12913-017-2125-3

For peer review only

For peer review only - http://bmjopen.bmj.com/site/about/guidelines.xhtml

52 Mallick L, Wang W, Temsah G. A comparison of summary measures of quality of service and quality of care for family planning in Haiti, Malawi, and Tanzania. Published Online First: 1 August 2017.https://dhsprogram.com/publications/publication-mr20-methodological-reports.cfm (accessed 17 May 2019).

53 Jackson EF, Siddiqui A, Gutierrez H, *et al.* Estimation of indices of health service readiness with a principal component analysis of the Tanzania Service Provision Assessment Survey. *BMC Health Serv Res* 2015;**15**:536. doi:10.1186/s12913-015-1203-7

54 Leslie HH, Zhou X, Spiegelman D, *et al.* Health system measurement: Harnessing machine learning to advance global health. *PLOS ONE* 2018;**13**:e0204958. doi:10.1371/journal.pone.0204958

55 Kruk ME, Chukwuma A, Mbaruku G, *et al.* Variation in quality of primary-care services in Kenya, Malawi, Namibia, Rwanda, Senegal, Uganda and the United Republic of Tanzania. *Bull World Health Organ* 2017;**95**:408–18. doi:10.2471/BLT.16.175869

56 Zurovac D, Guintran J-O, Donald W, *et al.* Health systems readiness and management of febrile outpatients under low malaria transmission in Vanuatu. *Malar J* 2015;**14**:489. doi:10.1186/s12936-015-1017-4

57 Akachi Y, Kruk ME. Quality of care: measuring a neglected driver of improved health. *Bull World Health Organ* 2017;**95**:465–72. doi:10.2471/BLT.16.180190

58 Leslie HH, Fink G, Nsona H, *et al.* Obstetric Facility Quality and Newborn Mortality in Malawi: A Cross-Sectional Study. *PLoS Med* 2016;**13**:e1002151. doi:10.1371/journal.pmed.1002151

59 Leegwater A, Wong W, Avila C. A concise, health service coverage index for monitoring progress towards universal health coverage. *BMC Health Serv Res* 2015;**15**:230. doi:10.1186/s12913-015-0859-3

60 Perez-Haydrich C, Warren JL, Burgert CR, *et al.* Guidelines on the use of DHS GPS data. Published Online First: 2013.https://dhsprogram.com/publications/publication-SAR8-Spatial-Analysis-Reports.cfm (accessed 14 Sep 2018).

61 FAQ - UNICEF MICS. http://mics.unicef.org/faq (accessed 19 Jun 2019).

62 Bliss RL, Katz JN, Wright EA, *et al.* Estimating Proximity to Care: Are straight line and zipcode centroid distances acceptable proxy measures? *Med Care* 2012;**50**:99–106. doi:10.1097/MLR.0b013e31822944d1

63 Jones SG, Ashby AJ, Momin SR, *et al.* Spatial implications associated with using Euclidean distance measurements and geographic centroid imputation in health care research. *Health Serv Res* 2010;**45**:316–27.

64 Healy MA, Gilliland JA. Quantifying the magnitude of environmental exposure misclassification when using imprecise address proxies in public health research. *Spat Spatio-Temporal Epidemiol* 2012;**3**:55–67. doi:10.1016/j.sste.2012.02.006

For peer review only

For peer review only - http://bmjopen.bmj.com/site/about/guidelines.xhtml

65 Nesbitt RC, Gabrysch S, Laub A, *et al.* Methods to measure potential spatial access to delivery care in low- and middle-income countries: a case study in rural Ghana. *Int J Health Geogr* 2014;**13**:25. doi:10.1186/1476-072X-13-25

66 Skiles MP, Burgert CR, Curtis SL, *et al.* Geographically linking population and facility surveys: methodological considerations. *Popul Health Metr* 2013;**11**:14. doi:10.1186/1478-7954-11-14

67 Turner A, Angeles G, Tsui A, *et al.* Sampling Manual for Facility Surveys for Population, Maternal Health, Child Health and STD Programs in Developing Countries, July 2001 — MEASURE Evaluation Manual Series, No. 3. 2001.https://www.measureevaluation.org/resources/publications/ms-01-03 (accessed 17 May 2019).

68 Baker U, Peterson S, Marchant T, *et al.* Identifying implementation bottlenecks for maternal and newborn health interventions in rural districts of the United Republic of Tanzania. *Bull World Health Organ* 2015;**93**:380–9. doi:10.2471/BLT.14.141879

69 Marchant T, Nathan R, Jones C, *et al.* Individual, facility and policy level influences on national coverage estimates for intermittent preventive treatment of malaria in pregnancy in Tanzania. *Malar J* 2008;**7**:260. doi:10.1186/1475-2875-7-260

70 Winter R, Wang W, Florey L, *et al. Levels and Trends in Care Seeking for Childhood Illness in USAID MCH Priority Countries. DHS Comparative Reports No. 38.* Rockville, MD, USA: : ICF International 2015.

71 Wang W, Soumya A, Wang S, *et al. Levels and Trends in the Use of Maternal Health Services in Developing Countries. DHS Comparative Reports No. 26.* Calverton, MD, USA: : ICF Macro 2011.

72 Noor AM, Amin AA, Gething PW, *et al.* Modelling distances travelled to government health services in Kenya. *Trop Med Int Health TM IH* 2006;**11**:188–96. doi:10.1111/j.1365-3156.2005.01555.x

73 Tanser F, Gijsbertsen B, Herbst K. Modelling and understanding primary health care accessibility and utilization in rural South Africa: An exploration using a geographical information system. *Soc Sci Med* 2006;**63**:691–705. doi:10.1016/j.socscimed.2006.01.015

74 Delamater PL, Shortridge AM, Kilcoyne RC. Using floating catchment area (FCA) metrics to predict health care utilization patterns. *BMC Health Serv Res* 2019;**19**:144. doi:10.1186/s12913-019-3969-5

75 Tsoka JM, Le Sueur D. Using GIS to measure geographical accessibility to primary health care in rural South Africa: research in action. *South Afr J Sci* 2004;**100**:329–30.

76 Gething PW, Noor AM, Zurovac D, *et al.* Empirical modelling of government health service use by children with fevers in Kenya. *Acta Trop* 2004;**91**:227–37. doi:10.1016/j.actatropica.2004.05.002

77 Tanser F, Hosegood V, Benzler J, *et al.* New approaches to spatially analyse primary health care usage patterns in rural South Africa. *Trop Med Int Health* 2001;**6**:826–38. doi:10.1046/j.1365-3156.2001.00794.x

78 Oehlert GW. A Note on the Delta Method. *Am Stat* 1992;**46**:27–9. doi:10.1080/00031305.1992.10475842

For peer review only - http://bmjopen.bmj.com/site/about/guidelines.xhtml

79 Sauer SM, Pullum T, Wang W, *et al.* Variance estimation for effective coverage measures: A simulation study. *J Glob Health* 2020;**10**:010506. doi:10.7189/jogh-10-010506

80 Brook RH, McGlynn EA, Cleary PD. Measuring Quality of Care. *N Engl J Med* 1996;**335**:966–70. doi:10.1056/NEJM199609263351311

81 Larson E, Sharma J, Bohren MA, *et al.* When the patient is the expert: measuring patient experience and satisfaction with care. *Bull World Health Organ* 2019;**97**:563–9. doi:10.2471/BLT.18.225201

82 Brizuela V, Leslie HH, Sharma J, *et al.* Measuring quality of care for all women and newborns: how do we know if we are doing it right? A review of facility assessment tools. *Lancet Glob Health* 2019;**7**:e624–32. doi:10.1016/S2214-109X(19)30033-6

83 Chakraborty NM, Sprockett A. Use of family planning and child health services in the private sector: an equity analysis of 12 DHS surveys. *Int J Equity Health* 2018;**17**:50. doi:10.1186/s12939-018-0763-7

84 Akin JS, Hutchinson P. Health-care facility choice and the phenomenon of bypassing. *Health Policy Plan* 1999;**14**:135–51.

85 Bell G, Macarayan EK, Ratcliffe H, *et al.* Assessment of Bypass of the Nearest Primary Health Care Facility Among Women in Ghana. *JAMA Netw Open* 2020;**3**:e2012552. doi:10.1001/jamanetworkopen.2020.12552

For peer review only

For peer review only - http://bmjopen.bmj.com/site/about/guidelines.xhtml

Table 1. Summary of publications included in the review and contribution to the literature

AUTHOR	YEAR	COUNTRY	KEY METHOD CONTRIBUTION
**SUITABILITY OF HOUSEHOLD AND PROVIDER DATA FOR LINKING ANALYSES** • **Household data needed for linked estimates**			
Ayede **[8]**	2018	Nigeria	• Accuracy of maternal report of pneumonia symptoms measured through household survey
Blanc **[17]**	2016	Mexico	• Accuracy of maternal report of delivery / immediate PNC attendant measured through household survey
Blanc **[18]**	2016	Kenya	• Accuracy of maternal report of delivery / immediate PNC attendant measured through household survey
Carter **[15]**	2018	Zambia	• Accuracy of maternal report of care-seeking for child illness measured through household survey
Chang **[14]**	2018	Nepal	• Accuracy of maternal report of birthweight and preterm birth measured through household survey
D'Acremont **[10]**	2010	SSA	• Reduced proportion of fever cases that are malaria
Hazir **[9]**	2013	Pakistan & Bangladesh	• Accuracy of maternal report of pneumonia symptoms measured through household survey
Keenan **[12]**	2017	US	• Accuracy of maternal recall of birth complications
Shengelia **[7]**	2005	-	• Effect of true versus perceived intervention need on effective coverage estimation
Stanton **[16]**	2013	Mozambique	• Accuracy of maternal report of place of delivery measured through household surveys
Walker **[11]**	2013	-	• Issues with measurement of child diarrhea through household surveys
Wang **[19]**	2018	Multiple Regions	• Issues with provider categories and alignment between DHS and SPA surveys
Zimmerman **[13]**	2019	Ethiopia	• Reliability of maternal report of maternal and newborn birth complications
**SUITABILITY OF HOUSEHOLD AND PROVIDER DATA FOR LINKING ANALYSES** • **Provider data needed for linked estimates**			
Akachi **[57]**	2017	-	• Need for global benchmarks for quality<br>• Lack on data linking quality with health outcomes
Carter **[33]**	2018	Zambia	• Quality score for child health effective coverage
Chou **[39]**	2019	Multiple Regions	• Quality score for maternal and neonatal health effective coverage
Davis **[32]**	2006	High-income countries	• Agreement between provider self-assessment and observed quality
Diamond-Smith **[24]**	2016	Kenya & Namibia	• Association between maternal perception of care and measured structural and process quality

For peer review only - http://bmjopen.bmj.com/site/about/guidelines.xhtml

Fisseha **[51]**	2017	Ethiopia	• Internal consistency of structural and process quality indicator
Gabrysch **[45]**	2011	Zambia	• Quality score for labor and delivery effective coverage
Getachew **[25]**	2020	Ethiopia	• Association between caregiver perception of care and measured structural and process quality
Hoogenboom **[27]**	2015	Thai-Myanmar Border	• Agreement between facility records and observed care
Hrisos **[30]**	2009	High-income countries	• Systematic review of agreement between observed quality of care and provider self-report, patient-report, and/or chart review
Jackson **[53]**	2015	Tanzania	• PCA to reduce quality index
Joseph **[42]**	2020	Malawi	• Quality score for ANC nutrition effective coverage<br>• Association between quality-adjusted coverage and LBW
Kanyangarara **[37]**	2017	SSA	• Quality score for ANC effective coverage
Kruk **[55]**	2017	SSA	• Association between structural and process quality
Larson **[26]**	2014	Tanzania	• Association between maternal perception of care and service availability and respect<br>• Vignettes for measuring quality
Leegwater **[59]**	2015	-	• Association between UHC index and infant mortality and life expectancy at national level
Leslie **[58]**	2016	Malawi	• Association between quality of delivery care and neonatal mortality
Leslie **[41]**	2017	SSA	• Quality score for ANC, labor and delivery, sick child, and family planning effective coverage<br>• Association between structural and process quality
Leslie **[54]**	2018	Multiple Regions	• Performance of approaches for generating service readiness indices
Leslie **[46]**	2019	Mexico	• Quality score for ANC, labor and delivery, newborn, sick child, chronic conditions, and cancer treatment effective coverage
Lozano **[49]**	2006	Mexico	• UHC index using weighted vs simple average of indicators
Mallick **[52]**	2017	Haiti, Malawi & Tanzania	• Comparison of measures of family planning quality
Marchant **[35]**	2015	Ethiopia, Nigeria, & India	• Measurement of quality using "last delivery module"
Mboya **[36]**	2016	Tanzania	• mHealth tool to measure quality
MCSP **[23]**	2018	Multiple Regions	• Availability and quality of data captured through HMIS
Moucheraud **[48]**	2020	SSA and Haiti	• Systematic review of quality measures derived from SPA data

For peer review only - http://bmjopen.bmj.com/site/about/guidelines.xhtml

Munos [40]	2018	Cote D'Ivoire	• Quality score for ANC, labor and delivery, PNC, and child health effective coverage
Nesbitt [28]	2013	Ghana	• Quality score for labor and delivery and PNC effective coverage
Nguhiu [38]	2017	Kenya	• Quality score for ANC, labor and delivery, sick child, and family planning effective coverage
Nickerson [21]	2015	Multiple Regions	• Comparison of data collected through health facility assessments
Osen [29]	2011	Ghana	• Agreement between provider reported and observed surgical service quality
Peabody [31]	2000	US	• Agreement between vignettes, chart abstraction, and simulated client measures
Serván-Mori [47]	2019	Mexico	• Quality score for labor and delivery and newborn care effective coverage
Sheffel [22]	2018	Multiple Regions	• Summary of quality data collected through SPA and SPA
Sheffel [50]	2018	Haiti, Malawi, Tanzania	• Association between structural and process quality
Willey [44]	2018	Uganda	• Quality score for labor and delivery and newborn care effective coverage
Wilunda [34]	2015	Uganda	• Quality score for maternal and neonatal care effective coverage
Zurovac [56]	2015	Vanuatu	• Poor association between structural quality and clinical care in fever management
**IMPLICATIONS OF DESIGN OF EXISTING HOUSEHOLD AND PROVIDER DATA SOURCES COMMONLY USED IN LINKING ANALYSES** • **Household data**			
Bliss [62]	2012	USA	• Comparison of distance using centroid versus true location
Healy [64]	2012	Canada & UK	• Comparison of distance using centroids of varying areal groupings
Jones [63]	2010	US	• Comparison of distance using zip-code centroid versus true household location
Nesbitt [65]	2014	Ghana	• Comparison of straight-line distance, network distance, raster and network-based travel time distance measures using village versus compound centroid
Perez-Heydrch [60]	2013	-	• Effect of DHS cluster displacement on distance measures
Skiles [66]	2013	Rwanda	• Effect of DHS cluster displacement on estimates of service environment

For peer review only - http://bmjopen.bmj.com/site/about/guidelines.xhtml

**IMPLICATIONS OF DESIGN OF EXISTING HOUSEHOLD AND PROVIDER DATA SOURCES COMMONLY USED IN LINKING ANALYSES**<br>• **Provider data**			
Carter **[33]**	2018	Zambia	• Effect of excluding non-facility providers from sampling frame on effective coverage estimates
Munos **[40]**	2018	Cote d'Ivoire	• Effect of excluding non-facility providers from sampling frame on effective coverage estimates
Skiles **[66]**	2013	Rwanda	• Effect of facility sampling on estimates of service environment
Turner **[67]**	2001	-	• Limitations of SPA sampling design<br>• Approach for joint sampling of households and facilities for linking analyses
**IMPLICATIONS OF DESIGN OF EXISTING HOUSEHOLD AND PROVIDER DATA SOURCES COMMONLY USED IN LINKING ANALYSES**<br>• **Survey timing**			
Baker **[68]**	2005	Uganda & Tanzania	• Stability of facility diagnostic capacity over time
Marchant **[69]**	2008	Tanzania	• Stability of IPTp stocks
Wang **[71]**	2011	Multiple Regions	• Stability of maternal health care-seeking behaviors measured through household survey over time
Willey **[44]**	2018	Uganda	• Stability of facility infrastructure indicators for labor, delivery, and newborn care
Winter **[70]**	2015	Multiple Regions	• Stability of care-seeking for child illness behaviors measured through household survey over time
**IMPACT OF CHOICE OF METHOD FOR COMBINING HOUSEHOLD AND PROVIDER DATA**<br>• **Comparison of exact-match and ecological linking methods for estimating effective coverage**			
Carter **[33]**	2018	Zambia	• Comparison of exact-match and ecological linking methods in estimating effective coverage in sick child care
Munos **[40]**	2018	Cote d'Ivoire	• Comparison of exact-match and ecological linking methods in estimating effective coverage in ANC, labor and delivery, PNC, and sick child care
Willey **[44]**	2017	Uganda	• Comparison of exact-match and ecological linking methods in estimating effective coverage in ANC, labor and delivery, PNC, and sick child care
**IMPACT OF CHOICE OF METHOD FOR COMBINING HOUSEHOLD AND PROVIDER DATA**<br>• **Performance of measures of geographic proximity for ecological linking**			
Carter **[33]**	2018	Zambia	• Comparison of true-source of care for child illness to straight-line and road distance measures
Delamater **[74]**	2019	US	• Comparison of FCA, simple distance, and Huff distance measure against true utilization patterns

For peer review only - http://bmjopen.bmj.com/site/about/guidelines.xhtml

Gething [76]	2004	Kenya	• Comparison of Theissen boundaries and true utilization patterns
Munos [40]	2018	Cote d'Ivoire	• Comparison of true-source of care for ANC, labor and delivery, PNC, and child illness to straight-line and road distance measures
Noor [72]	2006	Kenya	• Comparison of true-source of care for child fever to closest by Euclidian and road distance
Tanser [77]	2001	South Africa	• Comparison of Theissen boundaries and true utilization patterns
Tanser [73]	2006	South Africa	• Comparison of typical source of care to closest by travel time
Tsoka [75]	2004	South Africa	• Comparison of Theissen boundaries and true utilization patterns
IMPACT OF CHOICE OF METHOD FOR COMBINING HOUSEHOLD AND PROVIDER DATA<br>• **Statistical challenges**			
Sauer [79]	2020	-	• Comparison of exact, parametric bootstrap, and delta method for estimating effective coverage variance
Wang [19]	2018	Multiple Regions	• Use of Delta method for estimating effective coverage variance
Willey [44]	2018	Uganda	• Use of Delta method for estimating effective coverage variance

For peer review only - http://bmjopen.bmj.com/site/about/guidelines.xhtml

Table 2. Table of linking approaches

APPROACH	METHOD
**EXACT-MATCH**	Link to specific reported source of care
**ECOLOGICAL**	Link to one or more providers based on geographic proximity or administrative association
**Geographic Proximity**	
• **Straight-line /Euclidean distance**	Closest by absolute (crow-flies) distance
• **Manhattan distance**	Closest by sum of horizontal and vertical distance between points on a grid (blockwise)
• **Minokowski distance**	Closest by weighted average of Euclidean and Manhattan distance
• **Road Distance**	Closest by distance along a road (line and joint) network
• **Raster-based travel time**	Closest by travel time between points on a continous grid surface with variable transit speed coefficients in each cell
• **Network-based travel time**	Closest by travel time along a road network accounting for variable speed and road conditions
• **Buffer**	All providers within a defined radius from household
• **Theissen Polygon**	Define catchment boundaries based on optimal distance between known providers
• **Kernel Density Estimation**	Define relative draw of providers over geographic area weighted by quality
• **Interpolated Surface**	Define continuous surface of provider access or quality by smoothing between provider point data
• **Floating Catchment Area**	Define catchments for known providers allowing for cross-border use (catchment overlap) and distance decay
**Administrative**	All providers within administrative unit boundaries

For peer review only

For peer review only - http://bmjopen.bmj.com/site/about/guidelines.xhtml

Table 3. Exact versus ecological linking estimates for select indicators across studies

	Willey Labor & Delivery Structural QoC		Carter Child Health Urban Structural QoC		Carter Child Health Rural Structural QoC		Munos Labor & Delivery Structural QoC		Munos Labor & Delivery Process QoC		Munos Child Health Structural QoC		Munos Child Health Process QoC	
	% (95% CI)	% Diff	% (95% CI)	% Diff	% (95% CI)	% Diff	% (95% CI)	% Diff	% (95% CI)	% Diff	% (95% CI)	% Diff	% (95% CI)	% Diff
**Exact-match**	9.86 (3.2-16.5)	REF	49 (43.6-54.5)	REF	60.3 (55.6-65.1)	REF	37.2 (30.5-43.9	REF	40.1 (32.9-47.3)	REF	22.9 (18.2-27.5)	REF	16.8 (12.8-20.8)	REF
**Ecological - Geographic**														
Absolute Distance							36.5 (29.5-43.5)	-1.9%	39.8 (32.2-47.5)	-0.7%	18.2* (12.3-24.1)	-20.5%	14.3 (9.1-19.6)	-14.9%
Absolute Distance & Provider Category[a]			49.1 (43.7-54.6)	0.2%	61.1 (56.3-65.9)	1.3%	37 (30.0-44.0)	-0.5%	39.6 (32.1-47.1)	-1.2%	20.8* (16.1-25.4)	-9.2%	16.5 (12.2-20.7)	-1.8%
Road Distance							36.8 (30.0-44.0)	-1.1%	40.4 (32.6-48.1)	0.7%	16* (10.9-21.1)	-30.1%	13.8 (8.5-19.1)	-17.9%
Road Distance & Provider Category[a]			48.7 (43.2-54.1)	-0.6%	58.8 (54.1-63.5)	-2.5%	37.5 (30.4-44.6)	0.8%	40.2 (32.5-47.9)	0.2%	20.2* (16.0-24.4)	-11.8%	16.5 (12.3-21.8)	-1.8%
Radius 5 km & Provider Category[a]			49.2 (43.7-54.7)	0.4%	59.4 (54.8-64.1)	-1.5%								
Radius 10 km – Unweighted[b]							35.3* (29.3-41.4)	-5.1%	39.1 (32.0-46.2)	-2.5%	18.8* (14.9-22.7)	-17.9%	15.7 (12.4-19.1)	-6.5%
Radius 10 km – Weighted[c]							37.5 (30.6-44.4)	0.8%	39.8 (32.5-47.1)	-0.7%	19.1* (15.1-23.0)	-16.6%	15.6 (12.1-19.1)	-7.1%
KDE - Single			71.8* (69.3-74.2)	46.5%	55* (50.4-59.6)	-8.8%								
KDE- Aggregate			74.3* (73.2-75.5)	51.6%	54.9* (50.4-59.5)	-9.0%								

**Ecological - Administrative**														
Facility Catchment & Provider Category[a]			49.1 (43.6-54.6)	0.2%	59.8 (55.1-64.5)	-0.8%								
Sub-District & Provider Category[a]			49.4 (43.9-54.9)	0.8%	57.9 (53.4-62.4)	-4.0%								
District - Unweighted[b]	4.7* (21.4-31.7)	-52.5%					34.9* (29.0-40.8)	-6.2%	39 (32.3-45.7)	-2.7%	17.8* (14.6-21.0)	-22.3%	21* (17.2-24.8)	25.0%
District - Unweighted[b] & Provider Category[a]	11.0 (3.8-18.2)	11.8%					37 (30.4-43.6)	-0.5%	39.7 (32.7-46.7)	-1.0%	20.3* (15.8-24.8)	-11.4%	17.4 (13.3-21.4)	3.6%
District - Weighted[c]							37.9 (31.3-44.4)	1.9%	40.7 (33.7-47.7)	1.5%	19.7* (16.1-23.2)	-14.0%	21.2* (17.4-25.0)	26.2%
District - Weighted[c] & Provider Category[a]							38.8* (31.9-45.7)	4.3%	40.8 (33.6-48.0)	1.7%	21.1* (16.4-25.8)	-7.9%	17.1 (13.1-21.2)	1.8%

[a] Ecological linking restricted to only providers within the category (type of outlet, managing authority, and facility level) reported by survey respondent

[b] Simple average of provider quality scores applied, not accounting for differentials in patient volume

[c] Provider quality scores weighted by provider utilization volume for relevant health area

For peer review only

For peer review only - http://bmjopen.bmj.com/site/about/guidelines.xhtml

Table 4. Summary of evidence related to methodological issues for linking analyses and related needs for future research

**Suitability of household and provider data for linking analyses**<br>• Need valid data on target population for the intervention, and suitable data on service contact/care-seeking<br>• Need provider data reflective of select aspects of quality of care, standardized indices, and clear interpretation of measures		
**Issue**	**Evidence**	**Action**
How valid are data on target population for interventions?	• Symptom/diagnosis-based conditions may be biased<br>• Rare conditions are not captured with sufficient sample	• Explore alternative methods for defining population in need (eg biomarkers, Bayesian modeling of disease probability)
How valid are data on care-seeking?	• Limited data suggest respondent able to identify type of provider but not type of health worker<br>• Inconsistent and sometimes poorly defined provider categories	• Align categories of care across data collection tools<br>• Validate care-seeking in more settings / health areas
How are quality of care data being collected & what are the limitations of these methods?	• Mostly through health facility surveys<br>• HMIS data not widely used – limited QoC data collected<br>• Alternative methods (record review, provider or client report, etc) correlate poorly with provision of services / process quality	• Assess validity of existing QoC measurement methods<br>• Assess availability/usability of HMIS data for EC estimation<br>• Develop and test new methods for assessing provision of care and experience of care
How are quality measures being constructed & what do we know about the performance of these indices?	• Mostly SPA / SARA structural data, limited indicators on provision or experience of care, EmONC signal functions<br>• Variable set of indicators used based on guidelines and standards<br>• Many methods for combining indicators have been tried<br>• Handful of studies comparing methods produced conflicting results	• Develop standardized and validated summary QoC measures
How well do measures of quality track with each other, clinical quality and/or health benefit?	• Limited evidence of weak or no association between 1) structural and process quality, 2) experience of care and provision of care, 3) measured quality and clinical care / health outcomes	• Standardize methods and terminology for defining and interpreting QoC measures to more accurately reflect role in the coverage cascade
**Implications of design of existing household and health provider data sources commonly used in linking analyses**<br>• DHS / MICS household location unknown, cluster location displaced and may introduce imprecision into ecological linking analyses		

For peer review only

• SPA / SARA often use sample of facilities and subsample of client-staff interactions that may not be representative of true service environment<br>• Household and provider surveys are sampled and conducted independently → data are typically temporally and geographically discordant		
**Issue**	**Evidence**	**Action**
Does imprecise DHS/MICS household location data affect ecological linking results?	• Handful of studies suggest minimal effect on geolinking results	• Assess impact of household versus cluster centroid location versus displaced centroid in ecological linking analyses in multiple settings
How does SPA / SARA sampling design affect estimates?	• 2 studies suggest impact of excluding non-facility providers is context specific<br>• Client-staff interactions sampled to be representative at same level as overall survey – not at facility level<br>• One study showed sampling of facilities resulted in moderate misclassification of service environment across linking methods<br>• Joint sampling method proposed in 2001 – over-sample providers around sampled household clusters	• Assess effect of provider sampling (vs census) on linked estimates<br>• Assess effect of within-facility sampling of healthworkers and client-healthworker observations<br>• Triangulate with other sources of facility data (e.g., HMIS) to take advantage of the greater detail of the SPA assessment with the bigger sample of the facility records<br>• Account for uncertainty in estimates based on the facility-level data (e.g., multilevel structure)<br>• Test alternative sampling methods to improve representativeness of provider survey sampling for clients and healthworkers<br>• Test joint sampling methods for EC estimation
How stable are indicators over time?	• Studies demonstrate moderate indicator variability over months/years<br>• No studies directly related to effect on linking analyses	• Assess stability of key provider and household indicators<br>• Develop and test methods to account for unstable estimates, including more frequent data collection methods (eg, through HMIS) if needed
**Impact of choice of method for combining household and provider data**<br>• Multiple approaches for combining data sets, each with strengths and limitations<br>• Exact-match linking based on specific source of care most precise but ecological linking based on geographic proximity or administrative unit is more feasible		

For peer review only - http://bmjopen.bmj.com/site/about/guidelines.xhtml

Issue	Evidence	Action
How do exact-match and ecological linking approaches compare?	• 3 studies found ecological methods produced estimates similar to exact-match under certain conditions in settings with high use of public providers<br>• Restricting analyses by source of care category and/or weight by utilization volume improved agreement with exact-match	• Assess performance of ecological methods in settings with greater variation in provider landscape, provider quality<br>• Define guidance, such as provider quality variation thresholds, for selection of linking method
How do different ecological linking methods and measures of geographic proximity perform?	• Similar results using straight-line, road distance, and travel time<br>• Variable performance of ecological methods in identifying true source of care / reported category of care	• Identify preferred measures of geographic proximity to use in linking analyses<br>• Create standard, accessible tools for conducting geolinking
What are the statistical challenges in combining data for effective coverage estimation?	• Most analyses derive estimate variance from household sampling error<br>• 2 papers used delta method, but no comparison to other methods<br>• Simulation found variance estimation using delta method performed better than household error alone or parametric bootstrapping	• Continue developing tools and approaches for estimating uncertainty around linked estimates

For peer review only - http://bmjopen.bmj.com/site/about/guidelines.xhtml

Figure 1. PRISMA Flow Diagram

For peer review only

For peer review only - http://bmjopen.bmj.com/site/about/guidelines.xhtml

PRISMA 2009 Flow Diagram

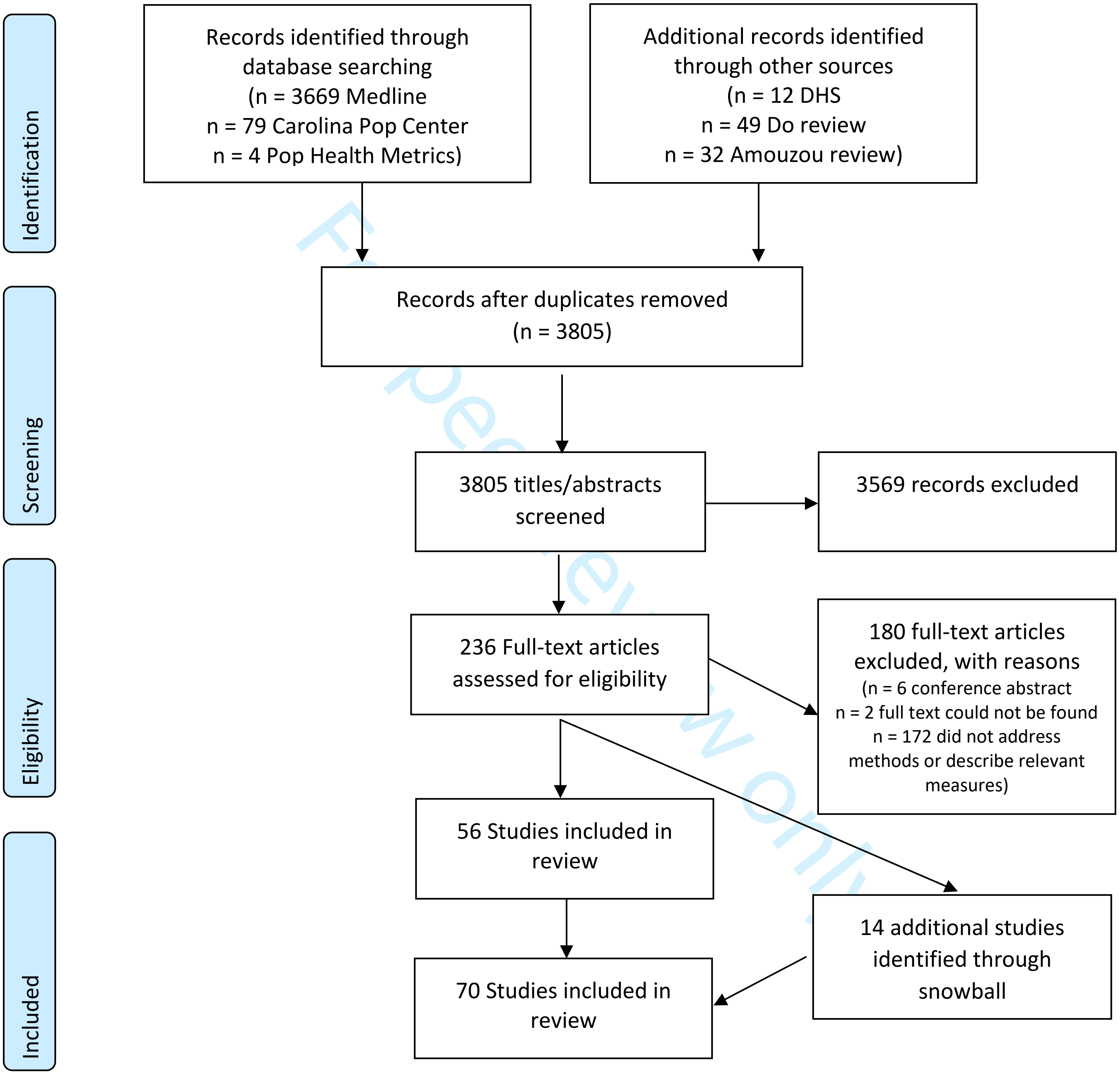

Supplemental File 1: Medline Search Terms

Term 1: Effective coverage (including related terms e.g. benchmarking, systems dynamics)

Term 2: Linked population-based and health provider analyses

Term 3: Population-based survey validity

Term 4: Health provider quality of care measurement

Term 5: Geospatial measurement of health provider utilization

Term 6: Date and Medline restriction

(Term 1 -OR- Term 2 -OR- Term 3 -OR- Term 4 -OR- Term 5) -AND- Term 6

Term 1:

(((coverage[Title/Abstract]) NOT (insurance[Title/Abstract])) AND (benchmark*[Title/Abstract])) OR (((coverage[Title/Abstract]) NOT (insurance[Title/Abstract])) AND (("systems dynamic framework"[Title/Abstract]) OR ("system dynamics framework"[Title/Abstract]) OR ("systems dynamics framework"[Title/Abstract]))) OR (((“universal health coverage”[Title/Abstract]) OR (UHC[Title/Abstract]) OR (“universal health care”[Title/Abstract])) AND ((coverage[Title/Abstract]) NOT (insurance[Title/Abstract])) AND ((metric*[Title/Abstract]) OR (measur*[Title/Abstract]) OR (score*[Title/Abstract]) OR (indicator*[Title/Abstract]) OR (variable*[Title/Abstract]) OR (index[Title/Abstract]) OR (indices[Title/Abstract]))) OR (("quality-adjusted") AND ((coverage) NOT (insurance[Title/Abstract]))) OR (("effective coverage"[Title/Abstract]) NOT (insurance[Title/Abstract])) OR

Term 2:

((((coverage) NOT (insurance)) AND ((link*[Title/Abstract]) OR (match*[Title/Abstract]) OR (combine*[Title/Abstract]) OR (merge*[Title/Abstract]) OR (attach*[Title/Abstract]) OR (join*[Title/Abstract]) OR (pair*[Title/Abstract]) OR (connect*[Title/Abstract]))) AND (("access to care") OR ("service quality") OR (quality of health care[MeSH Terms]) OR ("service readiness") OR ("service provision") OR ("service delivery") OR ("source of care") OR ("where care was sought") OR ("facility survey") OR ("provider survey") OR ("facility assessment") OR ("provider assessment") OR ("facility data") OR ("provider data") OR ("outlet survey") OR ("outlet assessment") OR ("outlet data")) AND (("service use") OR ("service utilization") OR ("care seeking") OR ("care-seeking") OR ("careseeking") OR ("doctor visit") OR ("clinic visit") OR ("facility visit") OR ("household survey") OR ("household data") OR ("household assessment") OR ("population survey") OR ("demographic survey") OR ("demographic and health survey") OR ("population-based"))) OR

Term 3:

((((("self report"[Title/Abstract]) OR ("self-report"[Title/Abstract]) AND (((maternal[Title/Abstract]) OR (newborn[Title/Abstract]) OR (child[Title/Abstract])) AND (health[Title/Abstract]))) OR ("maternal report"[Title/Abstract]) OR ("maternal recall"[Title/Abstract]) OR ("caregiver recall"[Title/Abstract]) OR ("caregiver report"[Title/Abstract]))) AND ((concordance[Title/Abstract]) OR (accuracy[Title/Abstract]) OR (valid*[Title/Abstract]))) OR

Term 4:

((((“structural quality”[Title/Abstract]) NOT (“structural equation”[Title/Abstract])) OR (infrastructur*[Title/Abstract]) OR (input*[Title/Abstract]) OR (readiness[Title/Abstract]) OR (capacity[Title/Abstract]) OR (“service provision”[Title/Abstract]) OR (“service environment”[Title/Abstract])) AND ((“quality of care”[Title/Abstract]) OR (process[Title/Abstract]) OR (“content of care”[Title/Abstract]) OR (“health outcome”[Title/Abstract]) OR (“health outcomes”[Title/Abstract]) OR ((“population health”[Title/Abstract]) AND (outcome*[Title/Abstract]))) AND ((metric*[Title/Abstract]) OR (measur*[Title/Abstract]) OR (indicator*[Title/Abstract]) OR (score*[Title/Abstract]) OR (variable*[Title/Abstract]) OR (index[Title/Abstract]) OR (indices[Title/Abstract])) AND (survey[Title/Abstract])) OR

Term 5:

((provider[Title/Abstract]) OR (facility[Title/Abstract]) OR (hospital[Title/Abstract]) OR ("source of care"[Title/Abstract])) AND ((geographic[Title/Abstract]) OR (spatial[Title/Abstract]) OR (GIS[Title/Abstract])) AND ((measur*[Title/Abstract]) OR (model*[Title/Abstract])) AND ((utilization[Title/Abstract]) OR ("usage"[Title/Abstract]) OR (distance[Title/Abstract]))) AND

Term 6:

(medline[sb]) AND ("2000/01/01"[PDat] : "2021/03/29"[PDat])

PRISMA 2009 Checklist

Supplemental File 2

Section/topic	#	Checklist item	Reported on page #
TITLE			
Title	1	Identify the report as a systematic review, meta-analysis, or both.	1
ABSTRACT			
Structured summary	2	Provide a structured summary including, as applicable: background; objectives; data sources; study eligibility criteria, participants, and interventions; study appraisal and synthesis methods; results; limitations; conclusions and implications of key findings; systematic review registration number.	2
INTRODUCTION			
Rationale	3	Describe the rationale for the review in the context of what is already known.	4
Objectives	4	Provide an explicit statement of questions being addressed with reference to participants, interventions, comparisons, outcomes, and study design (PICOS).	4
METHODS			
Protocol and registration	5	Indicate if a review protocol exists, if and where it can be accessed (e.g., Web address), and, if available, provide registration information including registration number.	N/A
Eligibility criteria	6	Specify study characteristics (e.g., PICOS, length of follow-up) and report characteristics (e.g., years considered, language, publication status) used as criteria for eligibility, giving rationale.	4
Information sources	7	Describe all information sources (e.g., databases with dates of coverage, contact with study authors to identify additional studies) in the search and date last searched.	4-5
Search	8	Present full electronic search strategy for at least one database, including any limits used, such that it could be repeated.	4 (supp. file)
Study selection	9	State the process for selecting studies (i.e., screening, eligibility, included in systematic review, and, if applicable, included in the meta-analysis).	4-5
Data collection process	10	Describe method of data extraction from reports (e.g., piloted forms, independently, in duplicate) and any processes for obtaining and confirming data from investigators.	5
Data items	11	List and define all variables for which data were sought (e.g., PICOS, funding sources) and any assumptions and simplifications made.	5
Risk of bias in individual studies	12	Describe methods used for assessing risk of bias of individual studies (including specification of whether this was done at the study or outcome level), and how this information is to be used in any data synthesis.	N/A
Summary measures	13	State the principal summary measures (e.g., risk ratio, difference in means).	N/A
Synthesis of results	14	Describe the methods of handling data and combining results of studies, if done, including measures of consistency (e.g., I^2) for each meta-analysis.	N/A

For peer review only

For peer review only - http://bmjopen.bmj.com/site/about/guidelines.xhtml

PRISMA 2009 Checklist

Section/topic	#	Checklist item	Reported on page #
Risk of bias across studies	15	Specify any assessment of risk of bias that may affect the cumulative evidence (e.g., publication bias, selective reporting within studies).	N/A
Additional analyses	16	Describe methods of additional analyses (e.g., sensitivity or subgroup analyses, meta-regression), if done, indicating which were pre-specified.	N/A
RESULTS			
Study selection	17	Give numbers of studies screened, assessed for eligibility, and included in the review, with reasons for exclusions at each stage, ideally with a flow diagram.	5
Study characteristics	18	For each study, present characteristics for which data were extracted (e.g., study size, PICOS, follow-up period) and provide the citations.	Table 1
Risk of bias within studies	19	Present data on risk of bias of each study and, if available, any outcome level assessment (see item 12).	N/A
Results of individual studies	20	For all outcomes considered (benefits or harms), present, for each study: (a) simple summary data for each intervention group (b) effect estimates and confidence intervals, ideally with a forest plot.	N/A
Synthesis of results	21	Present results of each meta-analysis done, including confidence intervals and measures of consistency.	N/A
Risk of bias across studies	22	Present results of any assessment of risk of bias across studies (see Item 15).	N/A
Additional analysis	23	Give results of additional analyses, if done (e.g., sensitivity or subgroup analyses, meta-regression [see Item 16]).	N/A
DISCUSSION			
Summary of evidence	24	Summarize the main findings including the strength of evidence for each main outcome; consider their relevance to key groups (e.g., healthcare providers, users, and policy makers).	11-12
Limitations	25	Discuss limitations at study and outcome level (e.g., risk of bias), and at review-level (e.g., incomplete retrieval of identified research, reporting bias).	12
Conclusions	26	Provide a general interpretation of the results in the context of other evidence, and implications for future research.	11-13
FUNDING			
Funding	27	Describe sources of funding for the systematic review and other support (e.g., supply of data); role of funders for the systematic review.	14

From: Moher D, Liberati A, Tetzlaff J, Altman DG, The PRISMA Group (2009). Preferred Reporting Items for Systematic Reviews and Meta-Analyses: The PRISMA Statement. PLoS Med 6(7): e1000097. doi:10.1371/journal.pmed1000097

For more information, visit: www.prisma-statement.org.

For peer review only
